# Biological self-protection inspired engineering of nanomaterials to construct a robust bio-nano system for environmental applications

**DOI:** 10.1126/sciadv.adp2179

**Published:** 2024-09-18

**Authors:** Nuo Xu, Xin Zhang, Pu-Can Guo, Dong-Hua Xie, Guo-Ping Sheng

**Affiliations:** CAS Key Laboratory of Urban Pollutant Conversion, Department of Environmental Science and Engineering, University of Science and Technology of China, Hefei 230026, China.

## Abstract

Nanomaterials can empower microbial-based chemical production or pollutant removal, e.g., nano zero-valent iron (nZVI) as an electron source to enhance microbial reducing pollutants. Constructing bio-nano interfaces is critical for bio-nano system operation, but low interfacial compatibility due to nanotoxicity challenges the system performance. Inspired by microorganisms’ resistance to nanotoxicity by secreting extracellular polymeric substances (EPS), which can act as electron shuttling media, we design a highly compatible bio-nano interface by modifying nZVI with EPS, markedly improving the performance of a bio-nano system consisting of nZVI and bacteria. EPS modification reduced membrane damage and oxidative stress induced by nZVI. Moreover, EPS alleviated nZVI agglomeration and probably reduced bacterial rejection of nZVI by wrapping camouflage, contributing to the bio-nano interface formation, thereby facilitating nZVI to provide electrons for bacterial reducing pollutant via membrane-anchoring cytochrome c. This work provides a strategy for designing a highly biocompatible interface to construct robust and efficient bio-nano systems for environmental implication.

## Teaser

Nanomaterials with microbial “coats” can better form highly compatible bio-nano interfaces with microorganisms.

## INTRODUCTION

Microbial-based processes for chemical production and environmental remediation have a promising application due to their low cost and low pollution (*1*–*3*). Nevertheless, the above processes are often characterized by low efficiency due to the limited metabolic rate of microorganisms (*4*). Nanomaterials can empower microorganisms due to their high surface area and high reactivity, making microbial-based chemical production or pollutant removal more efficient (*5*–*7*). Typical examples include that semiconductor nanomaterials provide electrons to microorganisms to intensify microbial conversion of CO_2_ to fuels or chemicals and nano zero-valent iron (nZVI) donates electrons to microorganisms to enhance microbial removal of pollutants (*8*, *9*). In these bio-nano systems, the formation of bio-nano interfaces is the key point for the efficient operation of the system, but low interfacial compatibility due to the biotoxicity of nanomaterials challenges the stability of bio-nano systems. Nanomaterials tend to cause impairment to cell membranes and cause oxidative damage to cells (*10*–*12*). In addition, metal ions released from metallic nanomaterials can also be toxic to microorganisms (*11*). Therefore, for bio-nano systems to move toward practical applications, there is an urgent need to design highly biocompatible bio-nano interfaces to improve system stability and sustainability.

**Fig. 1.**
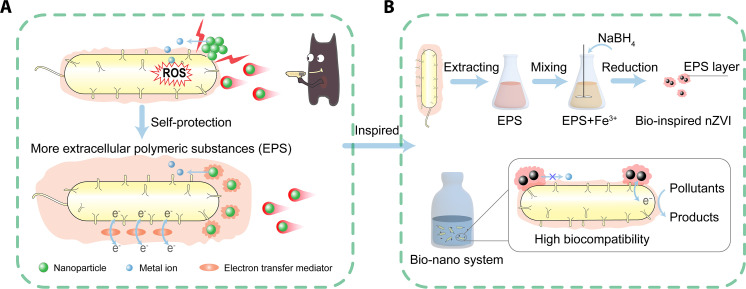
Schematic diagram of bio-inspired modification of nanomaterials using microbial EPS. (**A**) Mechanism of microbial mitigation of nanotoxicity by secreting EPS. (**B**) Scheme for the preparation of bio-inspired nZVI using EPS and construction of bio-nano system.

Through millions of years of evolution, microorganisms have developed a self-protection mechanism by secreting extracellular polymeric substances (EPS) to resist external stress such as nanoparticles, heavy metals, organic pollutants, etc. (*13*–*15*). EPS play a crucial role as interfacial biomacromolecules for microorganisms in contact with the external environment, mainly composed of proteins, polysaccharides, and humic acids, with diverse functional groups (*16*). EPS are the outermost protective layer of microbial cells, which can reduce direct contact between nanoparticles and cells, thus mitigating the membrane disruption induced by nanoparticles (Fig. 1A) (*17*). Furthermore, EPS secreted by microorganisms can be adsorbed on the surface of nanoparticles to form corona, and this EPS corona usually reduces the toxicity of nanoparticles by complexing nanoparticle-released ions (Fig. 1A) (*18*). EPS adsorption can also mitigate the aggregation of nanoparticles and improve their stability (Fig. 1A) (*19*). Besides, EPS can act as electron transfer media due to the presence of electron shuttles (e.g., cytochrome c and flavins) (Fig. 1A) (*20*, *21*). Inspired by these, modification of nanomaterials by microbial EPS is expected to improve the biocompatibility of nanomaterials and reduce their agglomeration as well as mediate the electron transfer between nanomaterials and microorganisms (Fig. 1B). Accordingly, we expect that the modification of nanomaterials using EPS secreted by microorganisms themselves allow us to construct a robust and efficient bio-nano system (Fig. 1B).

As a proof of concept, we constructed a bioinspried nZVI-enhanced microbial system as a model bio-nano system for pollutant removal. *Shewanella oneidensis* MR-1 (*S. oneidensis* MR-1), a typical widely distributed bacterium capable of transferring electrons outside the cell to reduce a wide range of pollutants, was selected as the model microorganism (*20*, *22*, *23*), and 3-nitrobenzenesulfonate (NBS), a common nitroaromatic compound in the industry, was selected as the target pollutant. We synthesized nZVI and EPS-modified bioinspired nZVI (nZVI_bio_) and constructed two bio-nano systems, i.e., bio-nZVI and bio-nZVI_bio_ systems for comparison. The stability and pollutant removal performance of these two systems were examined, and the underlying mechanism for the differences between the two systems was elucidated by analyzing the physicochemical properties and biocompatibility of these two nZVI as well as resolving the electron transfer pathway between nZVI and bacterial cells. This work is expected to provide an innovative approach for preparing highly biocompatible nanomaterials and designing robust and efficient bio-nano systems for environmental applications.

## RESULTS AND DISCUSSION

### Characterization of nanomaterials and construction of bio-nano systems

#### nZVI_bio_ and nZVI

**Fig. 2.**
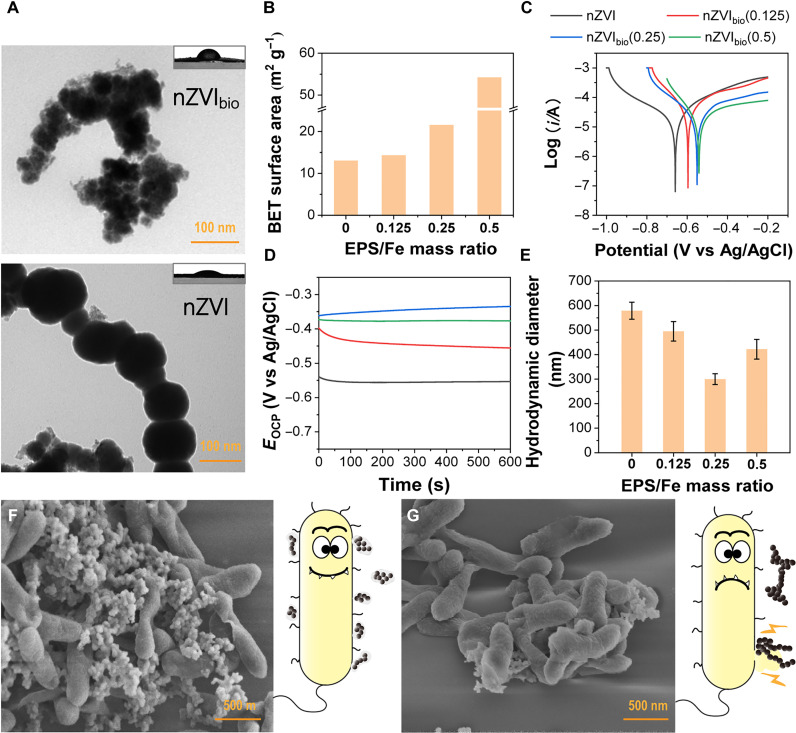
Characterization of nanomaterials and bio-nano systems. (**A**) TEM images and water contact angle of nZVI_bio_ and nZVI. Variation of the (**B**) BET surface area, (**C**) Tafel scans, (**D**) OCP, and (**E**) hydrodynamic diameter of nZVI_bio_ with varying EPS/Fe mass ratios from 0 to 0.5. SEM images of the (**F**) bio-nZVI_bio_ system and (**G**) bio-nZVI system along with the corresponding schematic diagram.

We used a borohydride reduction method to synthesize nZVI_bio_ and nZVI by adding a NaBH_4_ solution dropwise to the Fe^3+^ solution with or without EPS, respectively (*24*). X-ray photoelectron spectroscopy (XPS) revealed the presence of nitrogen in nZVI_bio_ but not in nZVI (fig. S1). X-ray diffraction (XRD) patterns exhibited Fe^0^ diffraction peaks in both nZVI_bio_ and nZVI (fig. S2). Transmission electron microscope (TEM) image of nZVI showed a characteristic chain-like structure with a diameter ranging from 50 to 100 nm, whereas nZVI_bio_ did not exhibit a beaded shape and was surrounded by a low-contrast corona (Fig. 2A). These observations signify successful modification of EPS on nZVI.

To ascertain the optimal modification level, we synthesized nZVI_bio_ with varying modification degrees by manipulating the mass ratio of EPS to Fe^3+^ ([EPS/Fe]_mass_) during the synthesis. The Brunauer-Emmett-Teller (BET) surface area of the nanomaterials exhibited an increase from 13.07 to 54.26 m^2^ g^−1^ as [EPS/Fe]_mass_ rose from 0 to 0.5 (Fig. 2B). The free corrosion potential of the nanomaterials increased from −0.66 to −0.54 V with [EPS/Fe]_mass_ increasing from 0 to 0.5, as depicted in the electrochemical polarization curve (Fig. 2C), suggesting that nZVI_bio_ exhibited a superior corrosion resistance compared to nZVI in aqueous environments (*25*). The open circuit potential (OCP) of nZVI_bio_ was higher than that of nZVI (Fig. 2D), confirming that nZVI_bio_ was more resistant to corrosion in water than nZVI, aligning with the result of Tafel scans (*26*). Dynamic light scattering (DLS) results showed a decrease in the hydrodynamic diameter as [EPS/Fe]_mass_ increased from 0 to 0.25, followed by an increase as [EPS/Fe]_mass_ reached 0.5 (Fig. 2E). This indicates that moderate EPS modification could optimize the dispersion of nanomaterials. Considering both corrosion resistance and dispersion, we chose nZVI_bio_ with [EPS/Fe]_mass_ = 0.25 for constructing the bio-nano system in subsequent experiments.

#### Bio-nano system

We constructed the bio-nano system by introducing nZVI or nZVI_bio_ into a bacterial culture medium, denoted as bio-nZVI and bio-nZVI_bio_, respectively. The bacteria exhibited a rod-shaped morphology, as revealed in the scanning electron microscope (SEM) image, which was consistent with the characteristic shape of *Shewanella* (fig. S3). We observed a substantial number of nanomaterials adhering to the bacterial surface in the bio-nZVI_bio_ system (Fig. 2F). Energy-dispersive x-ray spectroscopy (EDS) mapping confirmed that the materials that adhered to cells were iron nanoparticles (fig. S4). However, the attachment of nZVI to the bacterial surface was notably reduced in the bio-nZVI system compared with that in the bio-nZVI_bio_ system (Fig. 2G). TEM images further illustrated a notably higher likelihood of direct contact between bacterial cells and nZVI_bio_ (fig. S5, A to C). These findings suggest that EPS modification facilitated the attachment of nanomaterials to the cell surface to form bio-nano interfaces. We next used higher-magnification TEM to investigate if nanomaterials entered bacterial cells and observed that both nZVI_bio_ and nZVI were located outside bacterial cells (fig. S5, D and E), possibly due to the large size and interconnectedness of the nanomaterials. We used surface plasmon resonance (SPR) to quantitatively reveal differences in binding of the two nanomaterials, nZVI and nZVI_bio_, to bacteria. As shown in fig. S6, the binding amount of nZVI_bio_ to bacteria was higher compared to that of nZVI. The results showed that the apparent rate constant of nZVI_bio_ binding to *Shewanella* was 2.16 times that of nZVI. This further proved that our bioinspired modification effectively enhanced nZVI affinity to bacteria, consistent with TEM findings.

### NBS reduction by bio-nano systems

**Fig. 3.**
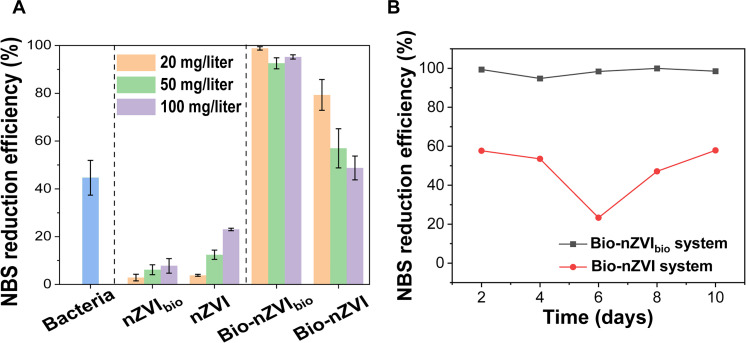
NBS reduction by the bio-nano systems. (**A**) Performance of NBS reduction by bacteria, nanomaterials, and bio-nano systems after a 48-hour treatment. (**B**) Sustainability of the bio-nano systems at a nanomaterial dose of 20 mg/liter.

Following the establishment of the bio-nano systems, we conducted a comparative analysis of NBS reduction by bacteria alone, nanomaterials alone, and bio-nano systems. Liquid chromatography results showed a gradual decrease in the peak area of NBS over time, with a new peak identified as the reduction product of NBS, 3-aminobenzenesulfonic acid (ABS), through qualitative analysis (fig. S7). Subsequently, we calculated the reduction efficiency of NBS (η_re_) based on ABS generation (fig. S8), given by

$$\eta_{\text{re}}\left(\%\right)=\frac{C_{t}}{C_{0}}\times 100$$ (1)

where *C_t_* is the concentration of ABS at time *t* (in M) and *C*_0_ is the initial concentration of NBS (in M). After a 48-hour treatment, the reduction efficiency of NBS by bacteria alone reached 44.7% (Fig. 3A and fig. S9). The reduction efficiencies of NBS by nZVI_bio_ (20, 50, and 100 mg/liter) were 2.9, 6.2, and 7.8%, respectively, which were lower than those by nZVI at the same doses, indicating that nZVI_bio_ had a lower reduction activity than nZVI (Fig. 3A). Nevertheless, the bio-nZVI_bio_ system exhibited a higher efficiency in reducing NBS compared to the bio-nZVI system. The reduction efficiencies of NBS in the bio-nZVI_bio_ system at material concentrations of 20, 50, and 100 mg/liter were 99.3, 93.6, and 98.6%, respectively; all of which were higher than those of the bio-nZVI system at the same nanomaterial dosages (Fig. 3A). Furthermore, the pollutant reduction efficiency of the bio-nZVI system gradually dropped with increasing nanomaterial dosage (<80%), whereas the bio-nZVI_bio_ system consistently maintained a high pollutant reduction efficiency (>90%) (Fig. 3A). These indicate the superior performance and stability of the bio-nZVI_bio_ system.

The reduction efficiencies of NBS by the bio-nZVI_bio_ system were 1.8 to 2.0 times of the sum of the reduction efficiencies of NBS by nanomaterials alone and by bacteria alone (fig. S10), indicating a synergistic effect between bacteria and nanomaterials in reducing NBS in the bio-nZVI_bio_ system. As for the bio-nZVI system, its NBS reduction efficiencies were 1.6, 1.0, and 0.7 times of the combined reduction efficiencies of NBS by bacteria alone and by nanomaterials alone at nZVI dosages of 20, 50, and 100 mg/liter, respectively (fig. S10). This indicates that the synergistic effect between nanomaterials and bacteria in the bio-nZVI system existed only at a low dosage of nZVI (20 mg/liter). With an increase in nZVI dosage to 50 mg/liter, the synergistic effect between bacteria and nanomaterials vanished, and a subsequent increase to 100 mg/liter led to an antagonistic effect. This could be attributed to the high toxicity of nZVI to bacteria (*11*). As the dosage increased, the inhibition on bacterial activity also increased gradually, resulting in a progressive decline in the pollutant removal performance of the bio-nZVI system. Nevertheless, EPS modification could substantially enhance the biocompatibility of nZVI, enabling bacteria to maintain a high activity and efficient pollutant removal performance even at high nanomaterial dosages.

To examine the differences in sustainability between the two systems, we performed a longer run of the bio-nano systems. As shown in Fig. 3B, the bio-nZVI_bio_ system exhibited stable operation for 10 days with a pollutant reduction removal efficiency of more than 95%. In contrast, the bio-nZVI system showed a low pollutant removal efficiency over 10 days of operation, and the system deteriorated even in the first few days. Therefore, the bio-nZVI_bio_ system was more robust than the bio-nZVI system and could operate efficiently for longer periods of time.

### Transformation of nanomaterials induced by bacteria in bio-nano systems

During the NBS reduction process, we investigated the transformation of nanomaterials within bio-nano systems using XPS and XRD. The Fe 2p spectra of nZVI_bio_ and nZVI revealed a dominant Fe(III) peak (711.2 and 724.8 eV) before the reaction (fig. S11A), indicating partial oxidation of the surface Fe^0^. Following the reaction, the Fe(III) peaks in nZVI_bio_ and nZVI clearly shifted to Fe(II) peaks (709.6 and 723.2 eV) in bio-nano systems (fig. S11A). Conversely, in the bacteria-free systems, nanomaterials did not show notable Fe(II) peaks after the reaction. These observations indicate that *S. oneidensis* MR-1, as a representative iron-reducing bacterium, played a role in facilitating the reduction of the outer oxide layer of nZVI, thus alleviating their passivation (*27*).

On the basis of the XRD spectra, we observed the transformation of nanomaterials within bio-nano systems into well-crystallized vivianite [(Fe_3_(PO_4_)_2_·8H_2_O] after a 48-hour reaction (fig. S11B). Vivianite is a monoclinic octahydrate ferrous phosphate crystal formed through the reaction of Fe(II) with phosphate ions (*28*). In the absence of bacteria, vivianite was also generated; however, the corresponding XRD peak was weaker compared to that with bacteria (fig. S11B). This indicates that bacteria facilitated the conversion of nanomaterials to vivianite, potentially due to the increased production of Fe(II) resulting from the reduction of the oxide layer induced by *S. oneidensis* MR-1. In addition, the similar XPS and XRD spectra of nZVI_bio_ and nZVI after a 48-hour reaction indicate that this biological modification method had a minimal impact on the transformation of nanomaterials induced by bacteria.

### Bacterial responses to nanomaterials in bio-nano systems

#### Changes in bacterial viability

**Fig. 4.**
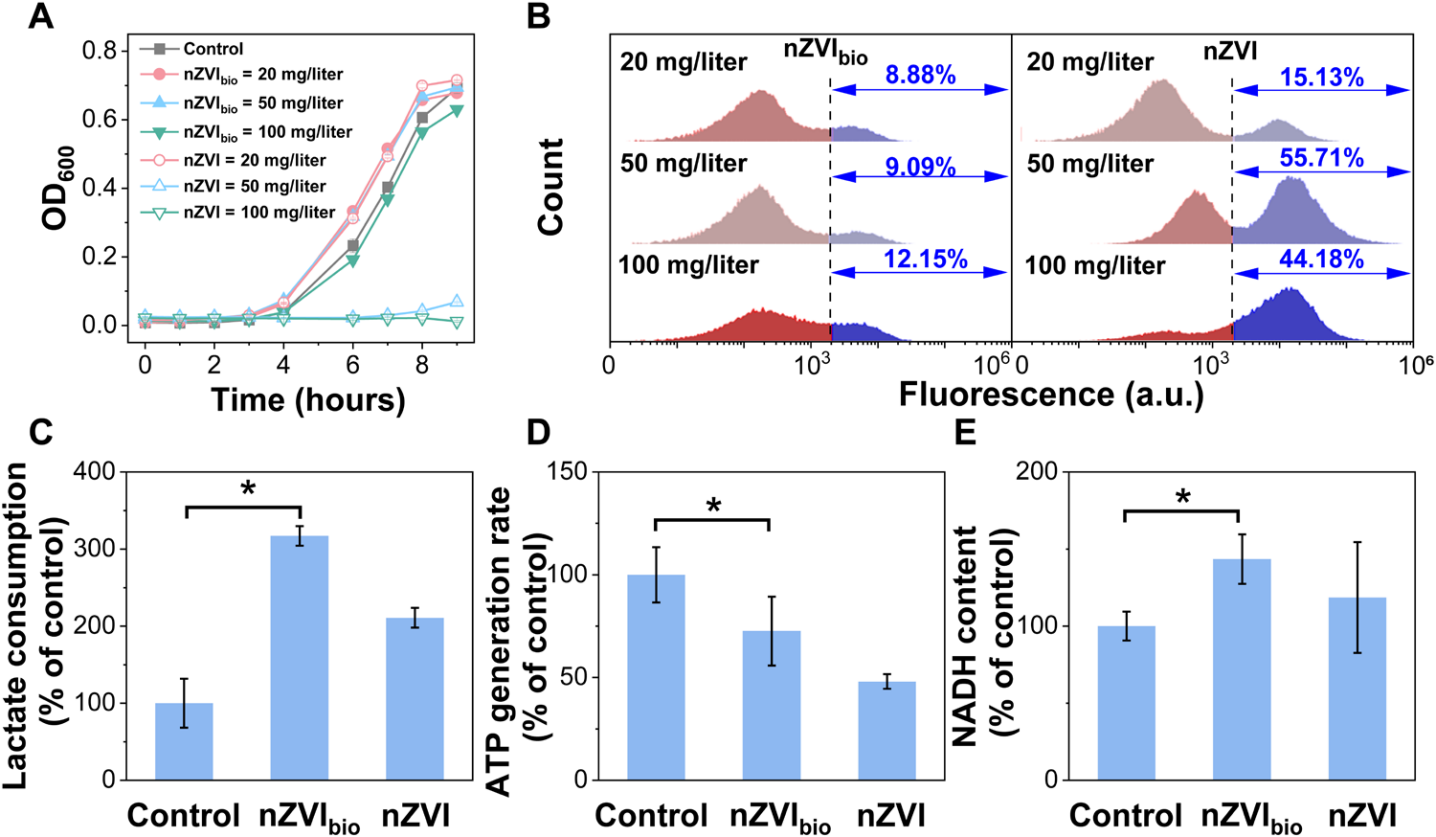
Variation of bacterial activity in bio-nano systems. (**A**) Growth recovery of *S. oneidensis* MR-1 after exposure to varying dosages of nZVI_bio_ and nZVI with an initial OD_600_ of 0.01. (**B**) Flow cytometry analysis showing the viabilities of *S. oneidensis* MR-1 after a 2-hour exposure to different dosages of nZVI_bio_ and nZVI. The red color represents the live cells, and the blue color represents the injured or dead cells. a.u., arbitrary units. (**C**) Lactate consumption by *S. oneidensis* MR-1 after a 48-hour exposure to nZVI_bio_ and nZVI (20 mg/liter), with an initial sodium lactate concentration of 18 mM. (**D**) ATP generation rate of *S. oneidensis* MR-1 after a 2-hour exposure to nZVI_bio_ and nZVI (20 mg/liter). The ATP concentration was determined using the BacTiter-Glo Microbial Cell viability assay kit, and the ATP generation rate was calculated through linear fitting of the ATP concentration to incubation time. (**E**) NADH content of *S. oneidensis* MR-1 after a 2-hour exposure to nZVI_bio_ and nZVI (20 mg/liter). Asterisk represents the significance at *P* < 0.05.

We recultivated the bacteria in bio-nano systems and monitored their growth to evaluate the impact of nanomaterials on their viability. Bacterial growth recovery exhibited a minimal impact across varying doses of nZVI_bio_, ranging from 20 to 100 mg/liter. For nZVI, bacterial resuscitation remained unaffected at a low dosage of 20 mg/liter; however, at dosages of 50 and 100 mg/liter, nZVI completely suppressed bacterial resuscitation (Fig. 4A). We next used nucleic acid staining and flow cytometry to quantify the proportion of injured or dead bacteria exposed to the nanomaterials. With an increase in nZVI_bio_ dosage, the proportion of injured or dead cells consistently remained at ~10% (Fig. 4B). Conversely, the addition of nZVI led to substantially higher percentages (15.1, 55.7, and 44.2%) of injured or dead cells at dosages of 20, 50, and 100 mg/liter, respectively (Fig. 4B). These results suggest that nZVI_bio_ had a lower biotoxicity compared to nZVI.

#### Changes in substrate utilization and ATP and NADH synthesis by bacteria

Generally, the metabolism of organic substrates can provide a carbon source, energy, and electrons for the growth of *S. oneidensis* MR-1 and pollutant reduction (*29*, *30*). We used high-performance liquid chromatography (HPLC) to monitor substrate consumption before and after NBS reduction experiments. In the bio-nZVI_bio_ and bio-nZVI systems, bacterial consumption of sodium lactate was 3.2 and 2.1 times of that of the control group without nanomaterials, respectively (Fig. 4C). This indicates that nanomaterials promoted bacterial utilization of organic substrates, and this promotion effect of nZVI_bio_ was more pronounced than that of nZVI. We then determined the rate of adenosine 5′-triphosphate (ATP) synthesis to assess the impact of nanomaterials on bacterial energy supply. The ATP synthesis rate decreased by 27.4% after exposure to nZVI_bio_ and by 51.9% after exposure to nZVI (Fig. 4D), suggesting that nZVI_bio_ had a smaller impact on bacterial energy supply than nZVI. Furthermore, the NADH [reduced form of nicotinamide adenine dinucleotide (oxidized form)] levels in bacterial cells were increased by 43.5 and 18.6%, respectively, with the addition of nZVI_bio_ and nZVI (Fig. 4E). This suggests that nanomaterials could provide electrons for bacteria to promote NADH synthesis, and the promotion of NADH synthesis by the bioinspired nanomaterials was greater than that by the pristine nanomaterials.

#### Changes in key genes’expression

**Fig. 5.**
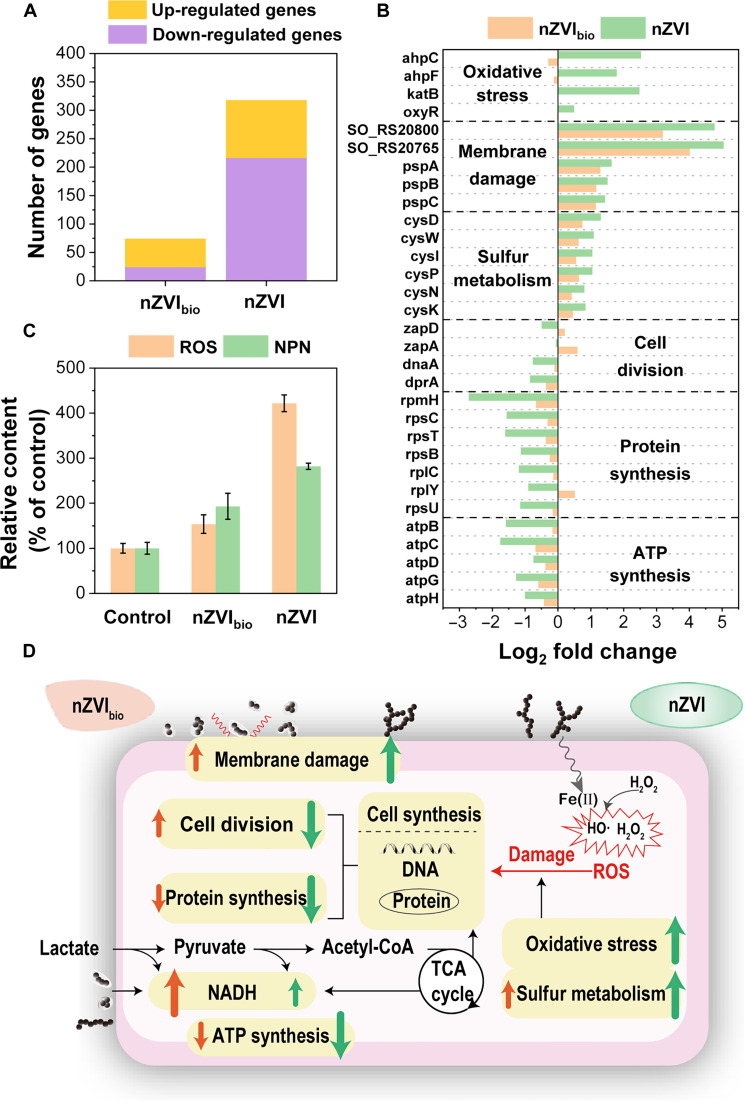
Transcriptomic analysis of bacterial responses to nanomaterial exposure. (**A**) Number of up-/down-regulated genes in bacteria after exposure to nZVI_bio_ or nZVI (20 mg/liter). (**B**) Fold change in the expression of various bacterial genes after exposure to nZVI_bio_ or nZVI (20 mg/liter). (**C**) Verification of intracellular ROS levels and membrane permeability in bacteria after a 2-hour exposure to nZVI_bio_ or nZVI (20 mg/liter). (**D**) Schematic diagram illustrating distinct stress responses of *S. oneidensis* MR-1 to nZVI_bio_ and nZVI in bio-nano systems. The red arrow represents the response of *S. oneidensis* MR-1 to nZVI_bio_, the green arrow represents the response of *S. oneidensis* MR-1 to nZVI, and the arrow length correlates with the response level. Acetyl-CoA, acetyl coenzyme A; TCA, tricarboxylic acid.

Exposure to nanomaterials can induce the stress response and detoxification repair in bacteria and influence various cellular processes such as cell division, biosynthesis, and energy supply (*10*, *31*). To investigate the physiological responses of bacteria to nanomaterials, we statistically analyzed the differential gene expression of bacteria by transcriptomic analysis in the bio-nZVI_bio_ and bio-nZVI groups in comparison to the control group without nanomaterials. The bio-nZVI_bio_ group exhibited 74 differentially expressed genes, whereas the bio-nZVI group showed 318 differentially expressed genes (Fig. 5A). This indicates that nZVI_bio_ had a substantially smaller impact on bacteria compared to nZVI. Furthermore, we selected some interesting differential genes for detailed investigation of the impact of nanomaterials on bacterial stress response and fundamental biological processes (Fig. 5B and table S1).

Previous studies have identified two primary toxic mechanisms of nZVI toward bacteria: (i) Fe(II) released from nZVI corrosion reacts with limited dissolved oxygen or endogenous H_2_O_2_ to produce reactive oxygen species (ROS), leading to intracellular accumulation of ROS and corresponding oxidative stress; and (ii) direct contact between nZVI and the cell membrane causes membrane damage, resulting in dysfunction of membrane proteins (*32*, *33*). As shown in Fig. 5B, increased expression of ROS-scavenging genes was observed after exposure to nZVI, especially in the genes encoding hydrogen peroxide enzymes (*ahpC*, *ahpF*, *katB*, and *oxyR*), which increased by 2.5-fold, 1.8-fold, 2.5-fold, and 0.5-fold, respectively.

Nevertheless, there was a minimal change in the expression of these genes in the nZVI_bio_-exposed group, indicating that nZVI_bio_ induced minimal ROS oxidative stress in bacteria. To validate the transcriptomic findings, we next measured intracellular ROS levels after exposure to nanomaterials. The ROS levels did not differ much between the bio-nZVI_bio_ group and the control group, while the bio-nZVI group produced 4.2 times more ROS than the control group (Fig. 5C), confirming that EPS modification reduced the oxidative damage to bacteria induced by nanomaterials (Fig. 5D).

In addition to oxidative stress, membrane damage constitutes another primary mechanism by which nanomaterials harm bacteria. We analyzed genes responsible for encoding membrane proteins and periplasmic space proteins (table S1). Upon the addition of nZVI_bio_ and nZVI, the gene (SO_RS20800) associated with membrane protein exhibited up-regulation by 2.2 and 3.8 times, while the gene (SO_RS20765) related to the periplasmic space protein displayed up-regulation by 3.0 and 4.0 times, respectively (Fig. 5B). Up-regulation of these genes aids in maintaining membrane stability and ensure normal membrane function (*34*). In addition, the phage shock protein (Psp) system responds to extracytoplasmic stress and can maintain membrane integrity and rigidity under stress (*34*, *35*). The expression levels of genes related to the Psp system, including *pspA*, *pspB*, and *pspC*, were notably up-regulated upon the addition of nZVI_bio_ or nZVI (Fig. 5B), further indicating membrane damage caused by nanomaterials. Notably, the up-regulation levels of genes related to membrane damage in the bio-nZVI_bio_ group were lower than those in the bio-nZVI group (Fig. 5, B and D). Subsequently, we used *N*-phenyl-1-naphthylamine (NPN) to measure the outer membrane permeability of bacteria after exposure to nanomaterials. The NPN fluorescence intensity of cells exposed to nZVI_bio_ was lower than that exposed to nZVI (Fig. 5C). These results proved that nZVI_bio_ induced less membrane damage compared to nZVI.

After encountering damages, many genes associated with detoxification and repair in bacteria were activated (table S1). Sulfur metabolism has been demonstrated to be crucial in maintaining the cellular antioxidant defense system (*36*, *37*). Upon exposure to nanomaterials, genes related to cysteine synthesis (*cysN*, *cysD*, *cysC*, *cysM*, *cysI*, and *cysK*) exhibited notable up-regulation in bio-nano systems (Fig. 5B). However, the expression of these genes in the bio-nZVI_bio_ group was lower than that in the bio-nZVI group, confirming that nZVI_bio_ caused less damage to bacteria and required less repair compared to the nZVI (Fig. 5D).

Regarding cell division, bacteria in the bio-nZVI system showed down-regulation of genes related to DNA replication (*dnaA* and *dprA*) and cell division proteins (*zapA* and *zapD*) (table S1) (*38*, *39*). In comparison, these genes were less down-regulated in the bio-nZVI_bio_ system, with some even showing up-regulation to varying degrees, such as *zapA* and *zapD* (Fig. 5B). In terms of protein biosynthesis, genes related to ribosomes (*rplC*, *rplY*, *rpmH*, *rpsB*, *rpsC*, *rpsT*, and *rpsU*) were involved in encoding proteins (table S1). Exposure to nZVI substantially down-regulated the expression of these genes, while exposure to nZVI_bio_ had a less effect on these gene expressions (Fig. 5B). Furthermore, the expression of genes related to ATP synthesis (*atpB*, *atpC*, *atpD*, *atpG*, and *atpH*) was down-regulated after exposure to nanomaterials, with the inhibitory effect of nZVI on ATP synthesis–related genes being much greater than that of nZVI_bio_ (Fig. 5B). In summary, the bioinspired nanomaterials through EPS modification showed a less impact on bacterial cell division, protein biosynthesis, and energy supply (Fig. 5D).

### Pathway of electron transfer from nanomaterials to bacteria

**Fig. 6.**
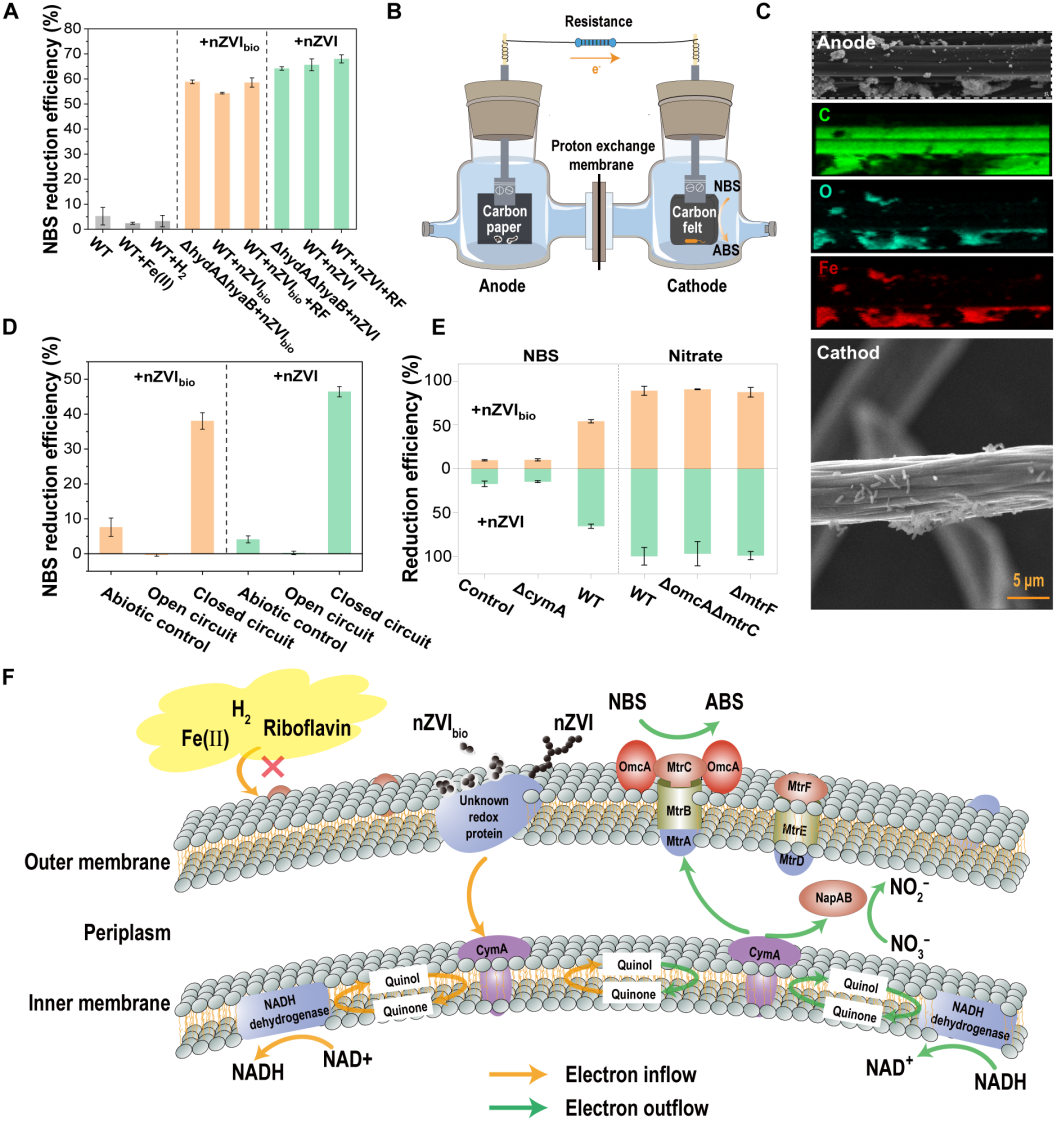
Pathway of electron transfer from nanomaterials to bacteria. (**A**) NBS reduction efficiency of various strains coupled with Fe(II), H_2_, riboflavin, nZVI_bio_, or nZVI in the absence of carbon source. WT, WT alone; WT + Fe(II), WT coupled with Fe(II); WT + H_2_, WT coupled with H_2_; WT + nZVI_bio_, WT coupled with nZVI_bio_; WT + nZVI_bio_ + RF, WT coupled with nZVI_bio_ and riboflavin; ΔhydAΔhyaB + nZVI_bio_, hydrogenase knockout strain coupled with nZVI_bio_; WT + nZVI, WT coupled with nZVI; WT + nZVI + RF, WT coupled with nZVI and riboflavin; ΔhydAΔhyaB + nZVI, hydrogenase knockout strain coupled with nZVI. (**B**) Schematic illustration of the two-chamber galvanic cell with nanomaterials loaded on carbon paper as the anode and *S. oneidensis* MR-1 loaded on carbon felt as the cathode. Titanium wire connected the electrodes, and a proton exchange membrane separated the two chambers_._ (**C**) SEM and EDS mapping images of the carbon paper and carbon felt. (**D**) NBS reduction efficiency of the abiotic group, the open circuit group, and the closed circuit group in the two-chamber galvanic cell. (**E**) NBS or nitrate reduction efficiency of different strains coupled with nZVI_bio_ or nZVI in the absence of carbon source. ΔcymA, CymA knockout strain; ΔomcAΔmtrC, OmcA and MtrC knockout strain; ΔmtrF, MtrF knockout strain. (**F**) Schematic mechanisms of electron transfer from extracellular nanomaterials to intracellular sites for NADH synthesis and from intracellular sites to the periplasm or extracellular space for pollutant reduction. NAD^+^, nicotinamide adenine dinucleotide (oxidized form).

In bio-nano systems, NBS could be reduced without the addition of a carbon source as an electron donor (Fig. 6A). Meanwhile, the reduction of NBS by nanomaterials alone was minimal, indicating that nanomaterials supplied electrons to support the reduction of NBS by bacteria. nZVI could give electrons directly, whereas in the case of nZVI_bio_, it was coated with an EPS corona, requiring electrons from the nanomaterials to pass through the EPS layer to reach the bacteria. Herein, we observed that EPS contained the electron shuttle cytochrome c, as evidenced by an ultraviolet-visible absorbance peak at 409 nm and a differential pulse voltammetry (DPV) peak at −155 mV (fig. S12) (*21*, *40*), which could mediate the transfer of electrons from nanomaterials to bacteria.

Interfacial electron transfer from nanomaterials to bacteria has been observed in many bio-nano systems (*41*). In previous studies, two potential mechanisms may be involved in the electron transfer from nZVI to *Shewanella*: (i) indirect electron transfer mediated by Fe(II) and H_2_ released from nZVI during corrosion and riboflavin secreted by bacteria and (ii) direct electron transfer mediated by redox proteins on the membrane of bacteria (*21*, *42*).

In the indirect electron transfer pathway, we explored the potential roles of Fe(II) and H_2_. When the nanomaterials in bio-nano systems were replaced by Fe(II) with the same amount of iron, the NBS reduction efficiency was almost equal to that of the control group with only bacteria (Fig. 6A and fig. S13). This implies that Fe(II) released by nZVI_bio_ or nZVI could not serve as an electron donor for bacteria in bio-nano systems. To determine if the H_2_ produced by nZVI_bio_ or nZVI corrosion could act as an electron donor for bacteria, we constructed a hydrogenase knockout strain ΔhydAΔhyaB. The NBS reduction by the ΔhydAΔhyaB strain was comparable to that of the wild-type (WT) strain with nZVI_bio_ or nZVI as the sole electron donor, indicating that H_2_ did not serve as an electron donor for bacteria to reduce NBS in the bio-nano systems. In addition, almost no NBS reduction by bacteria was observed when only H_2_ was used as the electron donor (Fig. 6A and fig. S13). Meanwhile, the monitoring H_2_ in the headspace was hardly changed (fig. S14), confirming that H_2_ could not serve as an electron donor for the NBS reduction by *S. oneidensis* MR-1. *S. oneidensis* MR-1 can release riboflavin extracellularly, facilitating electron transfer between bacteria and iron-based materials (*43*). However, the addition of riboflavin in bio-nano systems had no obvious promoting effect on the reduction of NBS (Fig. 6A and fig. S13). These results suggest that direct physical contact might be necessary for electron transfer from nZVI_bio_ or nZVI to *S. oneidensis* MR-1.

Direct electron transfer was verified using a two-chamber galvanic cell with nanomaterials loaded on carbon paper as the anode and *S. oneidensis* MR-1 loaded on carbon felt as the cathode (Fig. 6B). SEM images of the carbon paper and carbon felt revealed that the nanomaterials were loaded on the former, and many bacteria were attached to the latter (Fig. 6C). The two-chamber galvanic cell was separated by a proton exchange membrane to prevent the direct contact between bacteria and nanomaterials (*44*). Under the open circuit mode, no NBS reduction was observed in the cathode chamber without a carbon source, while the NBS was gradually reduced under the closed circuit mode (Fig. 6D and fig. S15). These results prove that nZVI_bio_ or nZVI directly provided electrons to the bacteria, thereby promoting the reduction of NBS. The potential monitoring of the galvanic cell also showed a notable potential difference (fig. S16), reflecting the spontaneous electron transfer from anode-loaded nanomaterials to the bacteria on the cathode.

*S. oneidensis* MR-1 typically uses the metal reduction pathway (i.e., “MtrCAB-CymA” or “MtrFDE-CymA” path) for electron transfer between the quinol pool in the cytoplasmic membrane and extracellular iron-based materials (*44*, *45*). To elucidate if *S. oneidensis* MR-1 acquired electrons from nZVI_bio_ or nZVI via the MtrCAB-CymA path or the MtrFDE-CymA path, mutant strains with the corresponding genes knocked out were constructed, namely, ΔcymA, ΔmtrF, and ΔomcAΔmtrC. The reduction of NBS by bacteria coupled with nZVI_bio_ or nZVI was almost inhibited after knocking out the inner membrane CymA (Fig. 6E and fig. S17A), indicating the essential role of CymA on the inner membrane in bacterial acquisition of electrons from nanomaterials. As NBS reduction by *S. oneidensis* MR-1 relies on outer membrane c-type cytochromes (c-Cyts), nitrate, which is reduced in the periplasm, was used as an indicator pollutant to investigate the role of outer membrane c-Cyts in electron transfer from nZVI_bio_ or nZVI to bacteria (Fig. 6E and fig. S18). When nanomaterials served as the sole electron donor, the reduction efficiencies of nitrate by the ΔomcAΔmtrC strain and ΔmtrF strain were comparable to those of the WT (Fig. 6E and fig. S17B), suggesting that bacterial electron acquisition from nZVI_bio_ or nZVI did not involve OmcA and MtrC and MtrF on the outer membrane. Electrons from nZVI_bio_ or nZVI might enter the membrane through unknown redox proteins on the outer membrane. In summary, nZVI_bio_ or nZVI bound to the outer membrane, transferring electrons into the periplasm via unidentified redox proteins, and subsequently, the electrons enter the intracellular space for bacterial synthesis of NADH through the cytoplasmic membrane of CymA (Fig. 6F).

### Mechanism for better stability and performance of the bio-nZVI_bio_ system over the bio-nZVI system

**Fig. 7.**
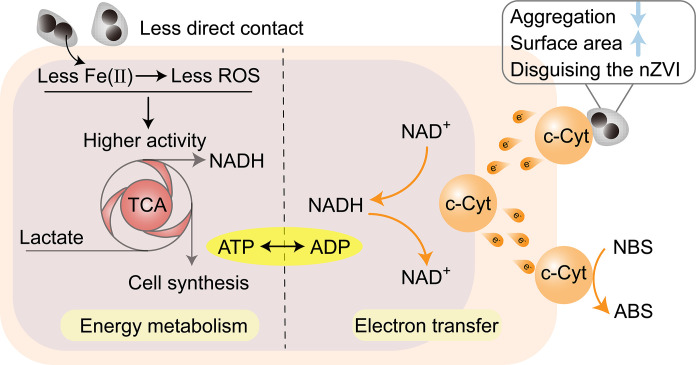
Mechanism diagram for high stability and high efficiency of the bio-nZVI_bio_ system. ADP, adenosine 5′-diphosphate.

EPS modification improved the biocompatibility of nZVI. Compared to nZVI, nZVI_bio_ exhibited a greater corrosion resistance in aqueous environments (Fig. 2C). In addition, the abundant negatively charged groups in EPS could complex nZVI-released Fe(II). Consequently, the nZVI_bio_ released less Fe(II) (fig. S19), weakening the bacterial intracellular Fenton reaction induced by Fe(II) and thus decreasing intracellular ROS production and the associated oxidative damage (Fig. 7) (*46*). Moreover, this biological modification mitigated the cell membrane damage induced by direct physical contact (Fig. 7). As such, nZVI_bio_ had a lesser impact on energy supply, protein biosynthesis, and cell division in bacteria compared to nZVI (Fig. 5D). The bio-nano system constructed with nZVI_bio_ exhibited higher stability.

nZVI tends to aggregate due to magnetism and surface tension, leading to reduced activity (*47*, *48*). EPS modification reduced the agglomeration of nZVI (Fig. 2E), enhancing its potential for electron utilization. In addition, EPS modification increased the specific surface area of nZVI (Fig. 2B). Meanwhile, EPS encapsulation probably disguised nZVI and reduced the rejection of foreign nZVI by bacteria (*49*, *50*). These facilitated easier binding of nZVI_bio_ to the bacterial surface. Given the direct electron transfer from nanomaterials to bacteria, more contact meant that more bio-nano interfaces could be formed for the nanomaterials to provide electrons to bacteria. Furthermore, due to the higher biocompatibility, the incorporation of nZVI_bio_ in the bio-nano system resulted in higher levels of NADH and ATP compared to those of nZVI (Fig. 4, C to E). Together, there were more energy and electrons available for bacteria in the bio-nZVI_bio_ system, supporting the reduction of pollutants (Fig. 7). Therefore, the bio-nZVI_bio_ system had superior performance in the pollutant reduction.

### Significance of this study

Compared with traditional chemical modifications to improve the compatibility of bio-nano interfaces, this biological modification strategy proposed in this study, inspired by the self-protection mechanism of microorganisms when coping with nanotoxicity, has the following advantages: (i) EPS are nontoxic, environmentally friendly, and readily available (*51*); (ii) EPS contain abundant and diverse groups (carboxyl, hydroxyl, amino, sulfhydryl, phosphoryl, etc.), enabling the modification of a broader range of nanomaterials (*52*); and (ii) for different bio-nano interfaces, the nanomaterials can be modified using EPS of the corresponding microorganisms, without considering the match between chemical modifiers and microorganisms (*53*, *54*).

Modifying nanomaterials using EPS secreted by microorganisms is anticipated to enhance the biocompatibility of various bio-nano interfaces, including typical semiconductor-bacteria interfaces and a traditional biological treatment reactor system (*55*). In a semiconductor-bacteria hybrid system, light-excited semiconductors generate holes that can oxidize and damage cell membranes. Furthermore, metal ions released from some metal-based semiconductors (e.g., CdS) cause toxicity to the bacteria, thereby resulting in the instability of semiconductor-bacteria interfaces (*56*, *57*). If a bioinspired semiconductor nanomaterial is prepared through modification with bacterially secreted EPS, the EPS can avoid direct contact between the nanomaterials and the cell membrane, slowing down the damage of the membrane by photo-generated holes. The reducing groups in the EPS can also provide electrons to quench the holes, promoting photogenerated carrier separation. Meanwhile, the introduction of iron-based nanomaterials undoubtedly brings technological innovation to traditional biological denitrification. Here, we try to apply this bioinspired modification strategy to an nZVI-enhanced denitrification system. The denitrification performance of a model denitrifying bacteria (i.e., *Paracoccus denitrificans*) coupled with nZVI and nZVI_bio_ was compared. The addition of nZVI had a certain toxic effect on denitrifiers, and the removal efficiency of total nitrogen even slightly decreased. However, the coupling of denitrifying bacteria with nZVI_bio_ could greatly enhance total nitrogen removal efficiency (fig. S20). This result proved that the bioinspired modification strategy we developed can be extended to other bio-nano systems.

In summary, the mechanism of biological coping with nanotoxicity offers innovative strategies for modifying nanomaterials to construct robust and efficient bio-nano systems, such as the EPS-modified strategy proposed in this study, and more and better bioinspired modification strategies need to be explored in the future.

## METHODS

### Bacterial strains and EPS extraction

WT *S. oneidensis* MR-1 and its mutant strains (ΔcymA, ΔmtrF, and ΔomcAΔmtrC) were kindly provided by L. Shi and Y.-C. Yong. The ΔhydAΔhyaB mutant was constructed using a method previously reported elsewhere (*58*). The EPS of *S. oneidensis* MR-1 was extracted through heat treatment (*21*). Breifly, *S. oneidensis* MR-1 cells were incubated in LB medium at 30°C with shaking at 200 rpm for 24 hours, then harvested by centrifugation (6500 rpm, 5 min), and washed three times with 0.9% NaCl (w/v) solution. The washed *S. oneidensis* MR-1 cells were resuspended and heated in a water bath at 60°C for 30 min. Last, *S. oneidensis* MR-1 cell suspensions were centrifuged (8500 rpm, 10 min) and the supernatant was filtered through a 0.22-μm filter to obtain the filtrate containing EPS.

### Synthesis of nZVI and nZVI_bio_

nZVI was synthesized using the sodium borohydride reduction method, and nZVI_bio_ was prepared through a one-step synthesis. Briefly, nZVI was synthesized by injecting 20 ml of a NaBH_4_ solution (76 g liter^−1^) into a three-necked flask containing 190 ml of an FeCl_3_·6H_2_O solution (10 g liter^−1^) under anaerobic conditions. nZVI_bio_ was synthesized by adding the NaBH_4_ solution to a mixture containing EPS and FeCl_3_·6H_2_O. The mass ratio between EPS and iron was precisely controlled at 0.125, 0.25, and 0.5, resulting in the synthesized nZVI_bio_ named as nZVI_bio_(0.125), nZVI_bio_(0.25), and nZVI_bio_(0.5), respectively. The synthesized materials were aged for 30 min before magnetic separation in a glove box and sequentially washed three times with deionized water and ethanol. Last, nZVI and nZVI_bio_ were dried in the glove box and ground for use.

### Construction of bio-nano systems and pollutant removal experiments

The washed *S. oneidensis* MR-1 cells were inoculated into an anaerobic, sterile mineral medium, as detailed in the Supplementary Text, with an initial optical density at 600 nm (OD_600_) of 0.1. nZVI_bio_ and nZVI were weighed, evenly dispersed in oxygen-free water, and subsequently introduced into the bacterial suspension to attain concentrations of 20, 50, and 100 mg/liter, followed by uniform oscillations. The resulting bio-nano systems, constructed using nZVI_bio_ and nZVI, were referred to as bio-nZVI_bio_ and bio-nZVI, respectively.

After the construction of bio-nano systems, batch experiments for pollutant removal were conducted in 60-ml serum bottles under anaerobic conditions. The bottles were supplemented with NBS at an initial concentration of 200 mg/liter to mimic industrial organic wastewater with a high pollutant concentration. Subsequently, the serum bottles were sealed using rubber stoppers and positioned in a shaker at 30°C with a constant shaking rate of 200 rpm. Periodic samples were collected to assess NBS concentrations, and all batch experiments were replicated three times.

### Evaluation of the toxicity of nZVI and nZVI_bio_ to bacteria

A *S. oneidensis* MR-1 cell resuspension solution was injected into a 30-ml anaerobic mineral medium to achieve an initial OD_600_ of 0.1. Subsequently, varying concentrations of nZVI_bio_ and nZVI (0, 20, 50, and 100 mg/liter) were added to initiate the exposure experiments. The viability of cells before and after a 2-hour exposure was assessed through resuscitation tests. Briefly, 1 ml of the exposed bacterial suspension was sampled and added to 20 ml of fresh LB medium, with bacterial growth monitored at various time points. Nucleic acid binding dyes, 4′,6-diamidino-2-phenylindole and propidium iodide, were used for cell staining. The stained cells were then analyzed by flow cytometry (CytoFLEX, Beckman Coulter Inc., United States) to quantify the proportion of injured or dead cells (details were given in the Supplementary Text).

Changes in intracellular ROS levels induced by exposure to nZVI_bio_ or nZVI were detected using 2',7'-dichlorodihydrofluorescein diacetate. Bacterial cell membrane permeability was evaluated using NPN. The ATP concentration was determined using the BacTiter-Glo Microbial Cell viability assay kit (G8230, Promega Corporation, United States), and the ATP biosynthesis rate was calculated by linear fitting of the ATP concentration to incubation time. The NADH content was determined by the NADH/NAD^+^ Assay Kit with WST-8 (Beyotime Biotechnology). Detailed information on the determination of intracellular ROS, cell membrane permeability, ATP, and NADH could be found in the Supplementary Text.

### Transcriptomic analysis of bacteria

To investigate the response of bacteria to the exposure of nZVI_bio_ or nZVI, we analyzed the transcriptomic profiles of *S. oneidensis* MR-1. Briefly, bacteria from the control group (without nanomaterials) and the experimental group [with nZVI_bio_ or nZVI (20 mg/liter); triplicate per group] were collected after a 2-hour exposure, washed three times with 0.1 M phosphate-buffered saline, and immediately inactivated under liquid nitrogen. The samples were then stored at −80°C, and subsequently, transcriptomic analysis was conducted at Novogene Bioinformatics Technology Co., Beijing, China (details were given in the Supplementary Text).

### Analyzing electron transfer pathway from nanomaterials to bacteria

To investigate the role of Fe(II) as an electron donor, the nanomaterials in bio-nano systems were replaced with Fe(II) of equivalent iron content, and the NBS reduction was monitored. To examine the role of H_2_ as an electron donor, the nanomaterials were replaced with H_2_, serving as electron donors for bacteria. In addition, we constructed the bio-nano system using the hydrogenase knockout strain (ΔhydAΔhyaB) and compared NBS reduction and H_2_ consumption in bio-nano systems constructed from the WT strain and ΔhydAΔhyaB strain. To investigate the possible role of riboflavin in mediating electrons from nanomaterials to bacteria, the bio-nano system was supplemented with riboflavin at levels comparable to bacterial secretion, and the change in NBS reduction was investigated.

To examine direct electron transfer from nanomaterials to bacteria, we designed a two-chamber galvanic cell in which the anode was carbon paper loaded with nanomaterials and the cathode was carbon felt, and the two chambers were separated by a proton exchange membrane. The cathode chamber was supplied with a mineral medium containing bacteria (OD_600_ = 0.2) and NBS (50 mg/liter), but no carbon source was added. Subsequently, the voltage and NBS reduction were monitored (details were given in the Supplementary Text).

To investigate the role of outer membrane c-Cyts in direct electron transfer, the bio-nano systems were constructed using the WT strain and specific mutants (ΔmtrF and ΔomcAΔmtrC). Because the bioreduction of NBS by *S. oneidensis* MR-1 is dependent on outer membrane c-Cyts, it is not appropriate to use NBS as an indicator pollutant to study the role of outer membrane c-Cyts in direct electron transfer. Instead, nitrate, which is reduced by nitrate reductase in the periplasmic space, was used as an indicator pollutant (*59*). To investigate the role of the inner membrane–anchored c-Cyts (i.e., CymA) in direct electron transfer, we compared the NBS reduction by the WT strain and ΔcymA strain using nanomaterials as the sole electron donor.

### Characterization

Details of various characterization techniques (SEM, TEM, SPR, XRD, XPS, DLS, BET surface area, contact angles, and zeta potential) were described in the Supplementary Text. Electrochemical characterizations, including OCP, Tafel curves, and DPV, were measured with a potentiostat (CHI760E, Chenhua Inc., China). The concentrations of NBS and ABS were determined by HPLC (model 1260, Agilent Inc., United States) (Supplementary Text). The concentration of NO_3_^−^ was determined by a spectrometer (UV2600, Shimadzu Co., Japan) (Supplementary Text).

## Supplementary Materials

### This PDF file includes:

Supplementary Text Figs. S1 to S20 Table S1 References
