# Supplementary material for: Biological self-protection inspired engineering of nanomaterials to construct a robust bio-nano system for environmental applications
Source: Sci Adv. 2024 Sep 18;10(38):eadp2179. doi: 10.1126/sciadv.adp2179 (PMC11409965; doi:10.1126/sciadv.adp2179)
Supplement: Supplementary file 1 — Supplementary Text Figs. S1 to S20 Table S1 References [file sciadv.adp2179_sm.pdf]

Science Advances
AAAS

Supplementary Materials for

Biological self-protection inspired engineering of nanomaterials to construct a robust bio-nano system for environmental applications

Nuo Xu *et al.*

Corresponding author: Xin Zhang, xzhang19@ustc.edu.cn; Guo-Ping Sheng, gpsheng@ustc.edu.cn

Sci. Adv. **10**, eadp2179 (2024)
DOI: 10.1126/sciadv.adp2179

This PDF file includes:

Supplementary Text
Figs. S1 to S20
Table S1
References

Supplementary Text

Chemicals and bacterial culture medium.

$FeCl_3 \cdot 6H_2O$, $FeCl_2 \cdot 4H_2O$, $NaBH_4$, NaOH, $NaNO_3$, ethanol (EtOH), riboflavin and sodium lactate were purchased from Sinopharm Chemical Reagent Co., Ltd. 3-nitrobenzenesulfonate (NBS) was purchased from Aladdin Chemicals Co., Ltd. and 3-aminobenzenesulfonic acid (ABS) was purchased from Aiyan Reagent Co., Ltd.

The *Shewanella* mineral medium contains (per liter): 2.154 g NaOH, 1.5 g NH_4Cl, 0.1 g KCl, 9.07 g PIPES buffer, 0.68 g $NaH_2PO_4 \cdot 2H_2O$ and 1 mL 1000× minerals stock solution *(60)*.

Bacterial viability test using flow cytometry.

Nucleic acid binding dyes, DAPI and PI, were used to test bacterial viability. The washed bacterial samples were reuspended in a PBS solution and added with 10 uL of PI and DAPI dyes, respectively. The samples were incubated in the dark at room temperature for 20 min and then washed twice with a PBS solution to eliminate excess dye. Finally, the samples were resuspended in a PBS solution and analysed using flow cytometry (CytoFLEX, Beckman Coulter, Inc., USA).

Measurement of intracellular ROS, membrane Permeability, ATP biosynthesis rate and NADH level.

Intracellular ROS. The intracellular ROS levels induced by nanomaterial exposure were detected by DCFH-DA. 4 μL DCFH-DA (25 mM) was added to 4 mL of the mineral medium containing bacteria. The mixed solution was oscillated and mixed at 30 °C for 30 min to load the probe. Exposure experiments were then initiated by adding different doses of $nZVI_{bio}$ or nZVI to each group. After a 15-minute exposure, each group was centrifuged at 7000 rpm for 3 min, and then the supernatant was removed, followed by washing twice with the mineral medium. Finally, the bacterial precipitates were resuspended in the mineral medium, and observed by a fluorescence spectroscopy at 525 nm with excitation at 488 nm.

Membrane Permeability Assay. S. oneidensis MR-1 treated with varying doses of $nZVI_{bio}$ and nZVI were collected and washed by sterile mineral medium. Then, NPN was added to reach a final concentration of 40 μg/L. The fluorescence intensity of NPN was measured with the excitation at 350 nm and emission at 420 nm.

ATP biosynthesis rate. The washed bacteria were inoculated into the sterile mineral medium with an initial OD_{600} of 0.2, and different doses of $nZVI_{bio}$ and nZVI were added to start the exposure experiment. 4 mL of mineral medium was collected after exposure for 15 min and mixed with 0.1mL fresh LB medium to incubate at 30°C with shaking. At 5, 15, 30, 45, and 60 min of incubation, a BacTiter-Glo™ Microbial Cell Viability Assay Kit (G8230, Promega Corporation) was used to determine the ATP content at the specific time according to the instructions. Briefly, 75 μL bacterial suspension was mixed with 75 μL BacTiter-Glo™ reagent, and the luminescence intensity was measured by a microplate reader after mixing for 5 min. The luminescence intensity of ATP standard solutions with different concentration gradients was also read. The ATP biosynthesis rate was calculated by linear fitting of ATP concentration over

the incubation time.

NADH level. The NADH content was determined through the WST-8 reaction by NADH/NAD^+ Assay Kit with WST-8 (Beyotime Biotechnology). The bacteria in bio-nano systems were centrifuged at 6000 rpm at 4°C for 5 min, suspended in a buffer solution, and then the NADH extract was added to lyse the cells. The lysed cells were centrifuged at 12000 rpm at 4°C for 10 min, and the supernatants were taken into 96-well plate after heating in a water bath. Color developing agent was added to the plate, and the absorbance was measured at 450 nm for determination.

Transcriptomic analysis.

Total RNA was isolated and extracted, and the total amount and integrity of RNA was assessed using an RNA Nano 6000 assay kit (Bioanalyzer 2100, California, America). Probes were used to eliminate rRNA in order to purify the total RNA and construct sequencing libraries. The reference genome and gene model annotation files (*S. oneidensis* MR-1) were obtained from NCBI [https://ftp.ncbi.nlm.nih.gov/genomes/all/GCA/000/012/525/GCA_000012525.1_ASM1252v1/] for read mapping using Bowtie2-2.2.3. DESeq R package (1.18.0) was used to analyze the differential expression of the control group and the experiment group.

Two-chamber galvanic cell experiment.

A proton exchange membrane separated the two cell chambers, with $nZVI_{bio}$ loaded carbon paper as the anode and carbon felt as the cathode. Titanium wire connected the electrodes. Both the anode and cathode chambers were filled with 100 mL of mineral medium without sodium lactate, and the cathode chamber additionally contained NBS (50 mg/L). The washed bacterial suspension was added to the cathode solution to achieve an initial OD_{600} of 0.2. During the reaction process, the two-chamber galvanic cell system was connected to a data collector to monitor the voltage difference between the cathode and anode. Periodic sampling of the cathode solution was performed to determine NBS and ABS concentration. The carbon paper and carbon felt were collected for SEM and EDS mapping after the reaction.

Characterization of nanomaterials and bio-nano systems.

The particle size distribution and zeta potential of $nZVI_{bio}$ and nZVI were measured using Malvern instrument (Zeta sizer Nano series, Malvern). The specific surface area was measured by a Surface Area and Porosity Analyzer (MicromeriticsTristar II 3020, USA). To determine the hydrophilicity of each material, equal mass powder (0.1 g) was compressed using a tablet press and water contact angles were measured with a contact angle goniometer (JC000D1, Powereach). Electrochemical characterizations, including open circuit potential (OCP) and Tafel scans, were measured in an oxygen-free 50 mM Na_2SO_4 solution using $nZVI_{bio}$ or nZVI loaded glassy carbon as the working electrode, platinum wire as the counter electrode, and Ag/AgCl (saturated KCl) as the reference electrode. The $nZVI_{bio}$ or nZVI loaded working electrode was prepared by a two-step tablet pressing method *(26)*. After recording OCP for 10 min, Tafel scanning was

performed with the three electrode system to record free corrosion potentials. In preparation for differential pulse voltammetry (DPV), 250 μL of EPS solution was combined with 250 μL of isopropyl alcohol and 10 μL of Nafion aqueous solution. This mixture was drop-casted onto a glassy carbon electrode and allowed to dry before being immersed in the electrolyte for testing *(21, 40)*. The parameters of DPV were as follows: E_i = -0.6 V; E_f = 0.4 V; amplitude, 50 mV; pulse width, 300 ms; and potential increment, 5 mV. The crystal structure and chemical composition of $nZVI_{bio}$ or nZVI before and after reaction were characterized by X-ray diffractometer (TTR III, Japan) and X-ray photoelectron spectroscopy (Thermo Scientific ESCALAB 250X, USA).

SPR analysis

Binding experiments of nano-iron particles on bacteria were performed with SPR device (BI-4500, Biosensing Instrument Co., USA). Each nanoparticles solution was injected at a flow rate of 25 μL/min, and with a contact time of 400 s. All measurements were carried out at 25°C. The concentration of nanoparticles was at a batch experimental dose of 50 mg/L. The binding process of nanoparticles on bacteria could be described by

$$\Delta I = I_{eq}(1 - e^{-tk_{obs}}) \qquad (1)$$

where ΔI *is* the change of the SPR signal after deducting the background, I_{eq} is the SPR signal of binding equilibrium and k_{obs} is the apparent rate constant (1/s).

Nanomaterials and bio-nano systems preparation protocols for TEM.

A suspension of bacteria and nanomaterials was briefly centrifuged and fixed with pre-cooled 2.5% glutaraldehyde at 4°C for 10 min. Subsequently, another centrifugation at 6000 rpm for 5 min was performed after decanting the culture solution. After removing the supernatant, the bacteria were fixed overnight at 4°C in a fresh fixing solution (5% glutaraldehyde and 4% paraformaldehyde). The fixing solution in the sample was sucked out and transferred to the recovery bottle. Subsequently, 0.1 M PB buffer (pH = 7.2) was added and rinsed three times at room temperature. Then the solution in the sample was sucked out and fixed at room temperature with 2% osmium tetroxide. The waste liquid was recycled, and the sample was rinsed three times with 0.1 M PB buffer (pH = 7.2) at room temperature. The washed samples were dehydrated with sequential treatment with 30, 50, 70, 80, 90, and 100% ethanol for 20 min. The samples were then infiltrated and embedded in Spurr's resin with propylene oxide (treatment with 1:1 and 1:3 of propylene oxide/Spurr's resin mixtures for 1 h and 3 h, and 100% Spurr's resin for 24 h). Finally, the polymerized blocks were placed on the top layer of the oven at 70°C and polymerized for over 8 h. The polymerized blocks were sectioned using an ultra-microtome (Leica UC7, Germany) and observed via a transmission electron microscopy (H-7650, Japan).

Bacteria and bio-nano systems preparation protocols for SEM.

Cells before and after treatment with $nZVI_{bio}$ or nZVI were collected via centrifugation. The obtained cells underwent three washes with sterile 0.85% NaCl and were fixed

overnight with 2.5% glutaraldehyde at room temperature. Next, the cells were collected by centrifugation and washed three times with 0.85% NaCl. The washed samples were dehydrated with sequential treatment with 30, 50, 70, 80, 90, and 100% ethanol for 20 min. The samples were sputtered with Au and characterized by a scanning electron microscope (GeminiSEM 500, USA).

Liquid and gas chromatography analysis.

The concentrations of NBS and ABS were determined by a high performance liquid chromatography (Model 1260, Agilent) with a C18 column (Agient Technologies, 5 μm, 4.6 × 250 mm). The mobile phase comprised a mixed solution of tetraethylammonium bromide (1 g/L) and methanol, with a ratio of 40:60 (v/v) and a flow rate of 0.5 mL/min. Detection of signals was set at 254 nm. The concentration of sodium lactate was determined by a high performance liquid chromatography (Model 1260, Agilent) with a hydrogen column (Hi-Piex H, 300 × 7.7 mm). Hydrogen concentration in the headspace of a 60 mL serum vial was detected using a gas chromatography (SP-6890, Lunan Corp).

Determination of NO_3^- concentration.

The concentration of NO_3^- was determined by a spectrometer (UV2600, Shimadzu). Briefly, 400 μL sample was added to a 5 mL centrifuge tube with 1.6 mL deionized water, followed by 16 μL of 0.2% sulfamic acid and 10 μL of 1 M HCl. The solution and standard product were spectrophotometrically analyzed for absorbance at 220 nm and 275 nm after thorough mixing. The corrected nitrate absorbance was obtained by subtracting twice the absorbance at 275 nm from the absorbance at 220 nm.

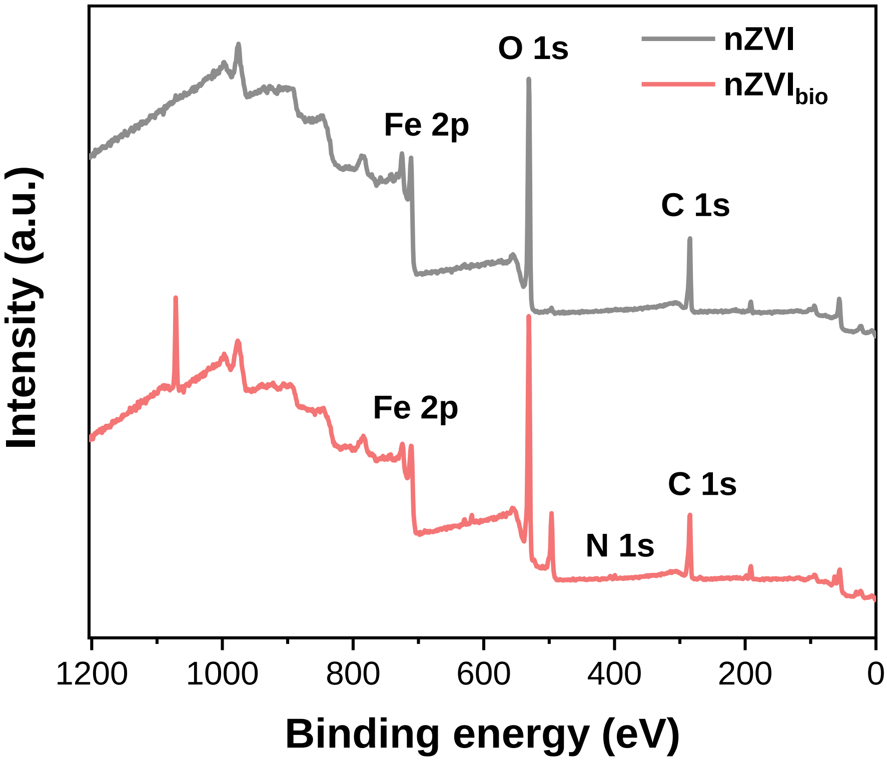

Fig. S1. The full-scan XPS spectra for $nZVI_{bio}$ and nZVI.

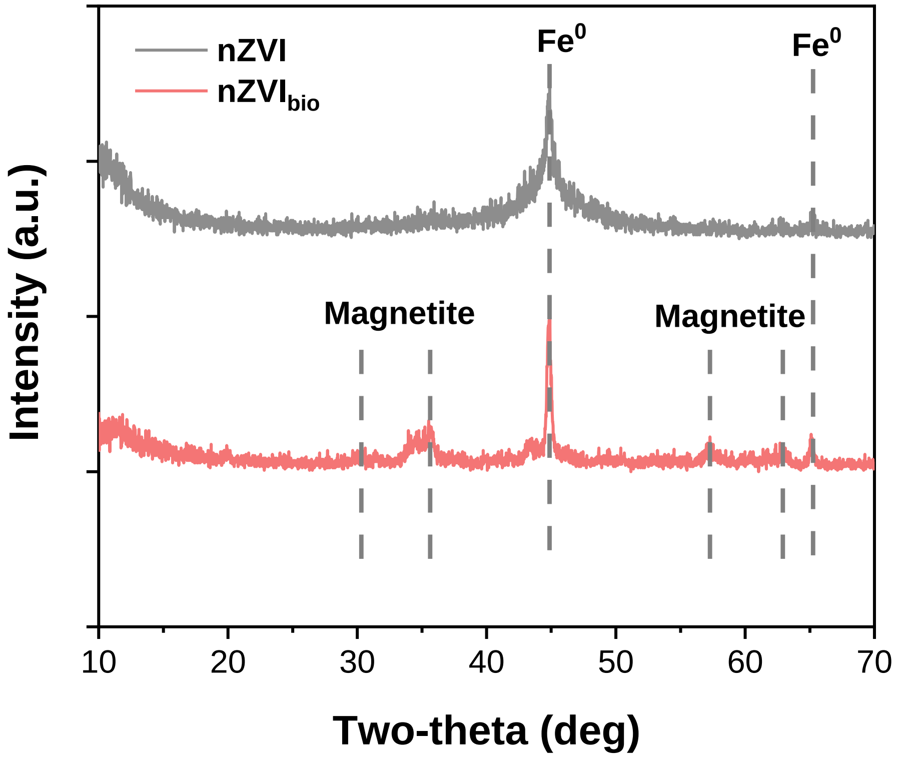

Fig. S2. XRD patterns of $nZVI_{bio}$ and nZVI.

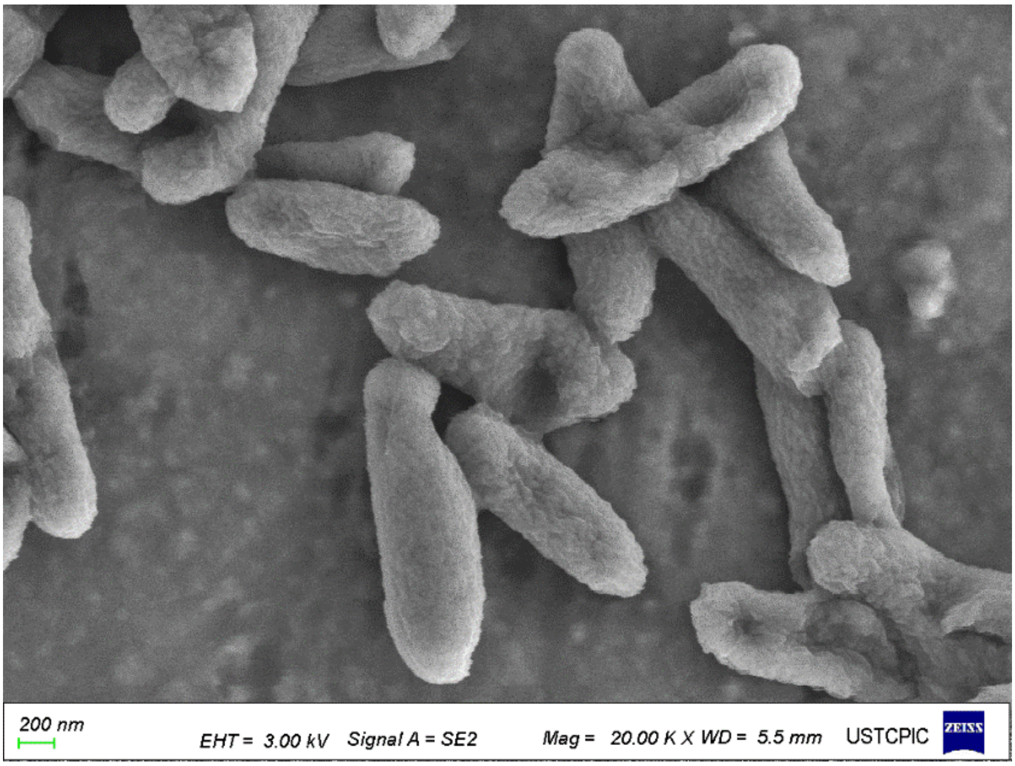

Fig. S3. SEM image of *Shewanella oneidensis* MR-1.

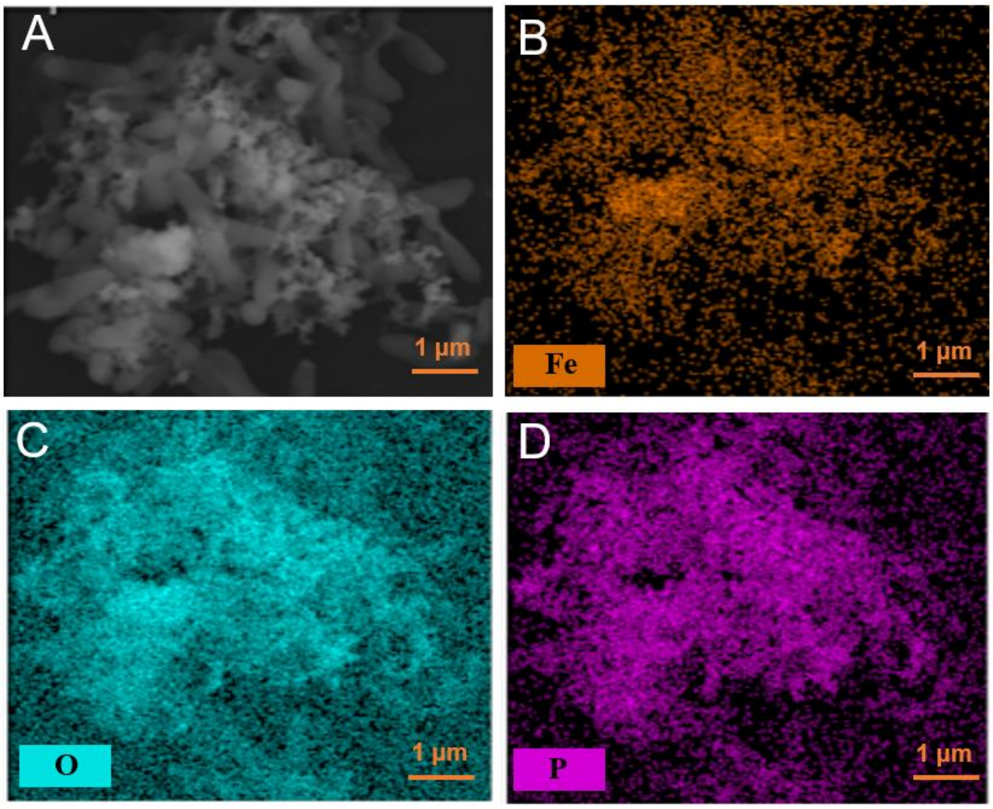

Fig. S4. Energy-dispersive x-ray spectroscopy (EDS) mapping of bio-nZVI$_{bio}$ system.

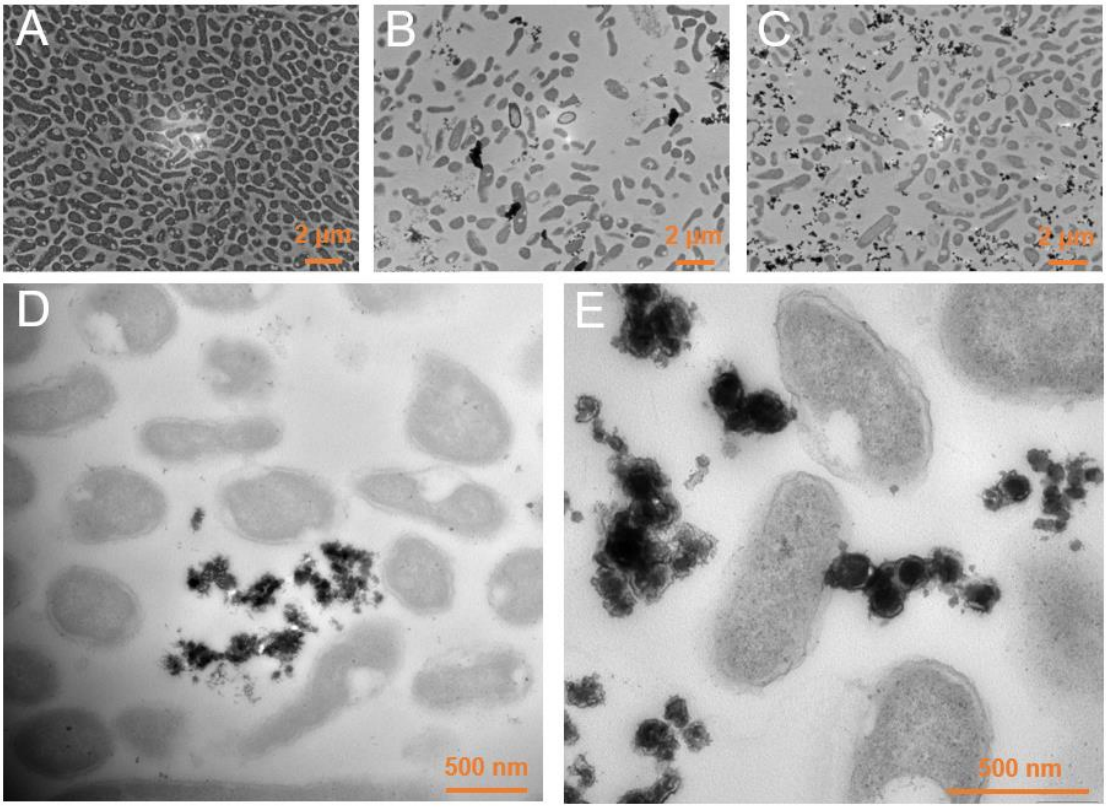

Fig. S5. TEM images of bio-nano systems. TEM images of (**A**) bacteria, (**B**) bio-nZVI system and (**C**) bio-$nZVI_{bio}$ system at the same magnification. TEM images of (**D**) bio-nZVI system and (**E**) bio-$nZVI_{bio}$ system at the higher magnification.

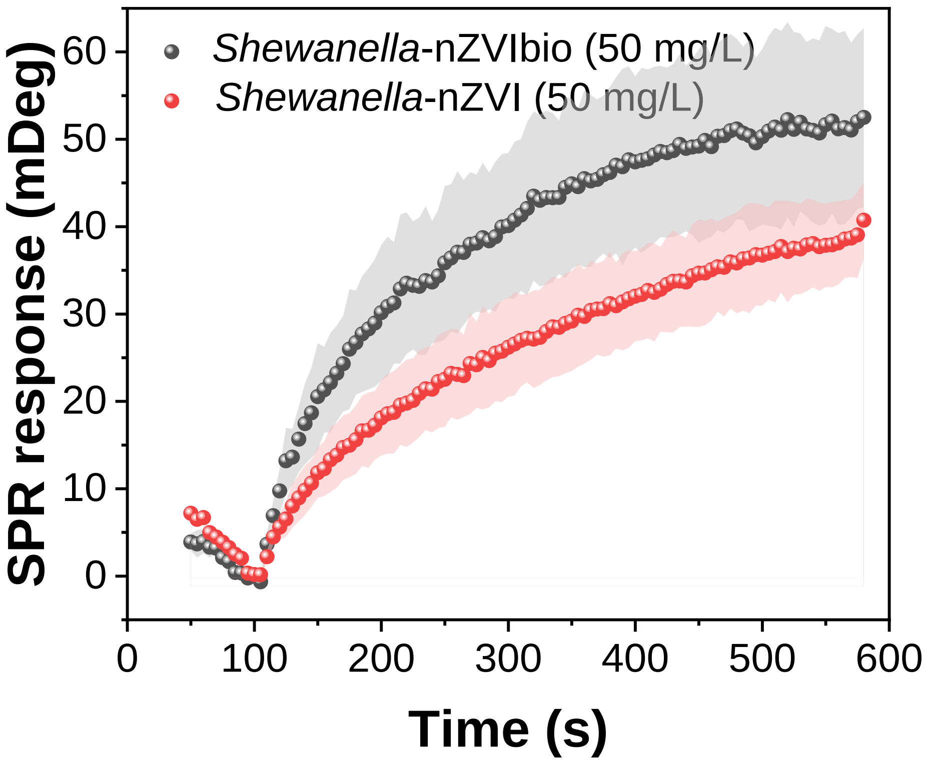

Fig. S6. SPR sensorgram of the binding of nZVI$_{bio}$ or nZVI (50 mg/L) to *Shewanella*.

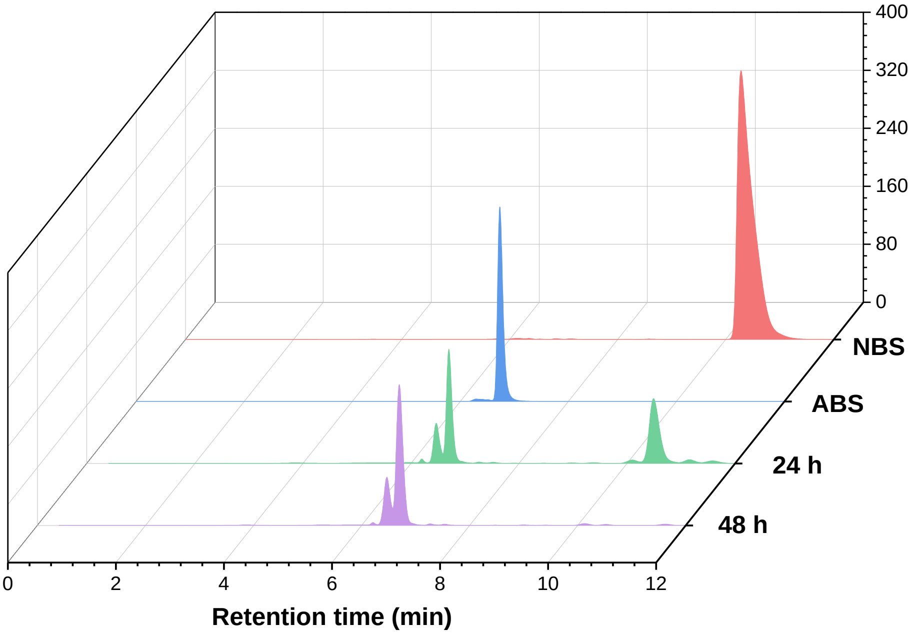

Fig. S7. HPLC results for NBS and its reduction products ABS.

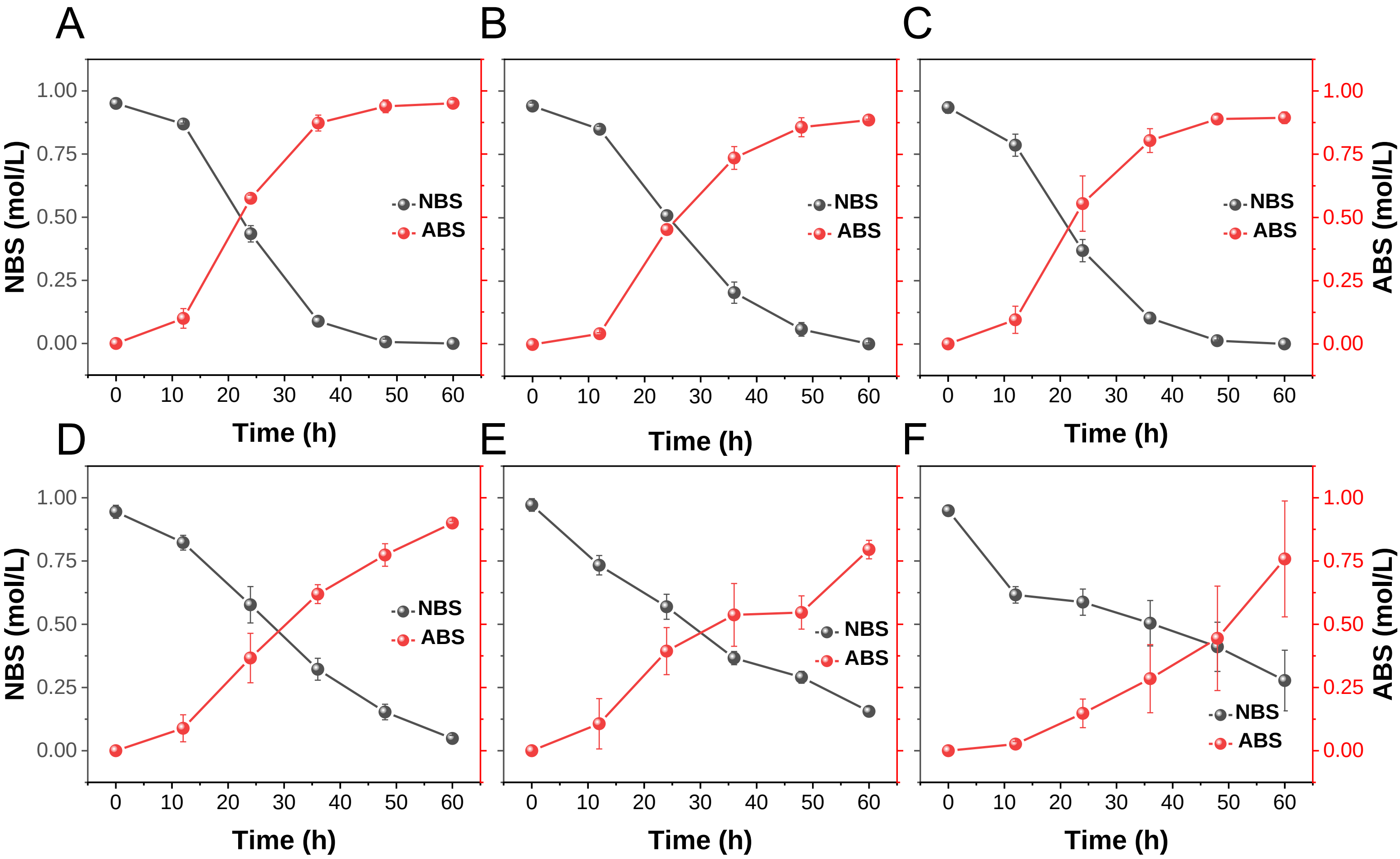

Fig. S8. Reduction of NBS by bio-nano systems with different material dosages. Changes in the concentrations of NBS and ABS in the bio-nano systems over time with addition of (**A**) 20 mg/L, (**B**) 50 mg/L and (**C**) 100 mg/L $nZVI_{bio,}$ as well as (**D**) 20 mg/L, (**E**) 50 mg/L and (**F**) 100 mg/L nZVI.

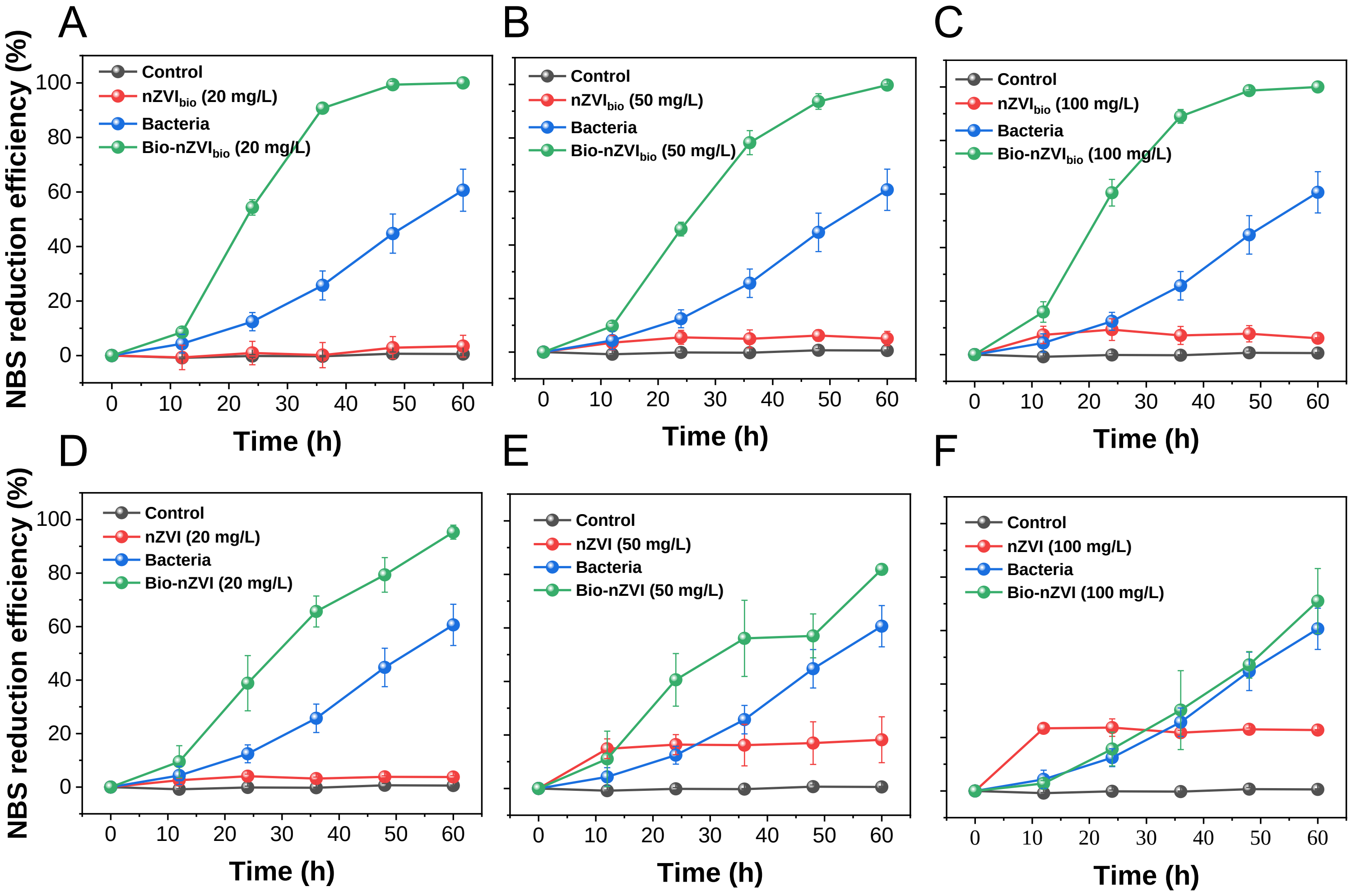

Fig. S9. Variations in the reduction efficiency of NBS by nanomaterials, bacteria or bio-nano systems over time.

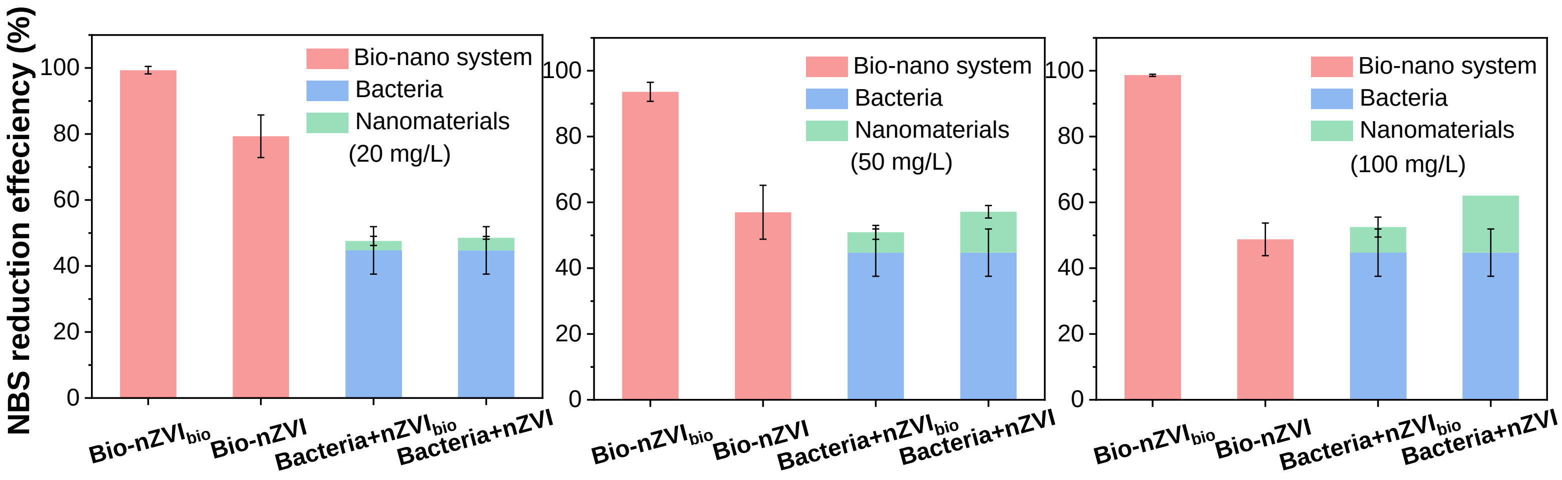

Fig. S10. Reduction efficiencies of NBS by bio-nano systems or the sum of the reduction efficiencies of NBS by nanomaterials alone and by bacteria alone after a 48-hour treatment.

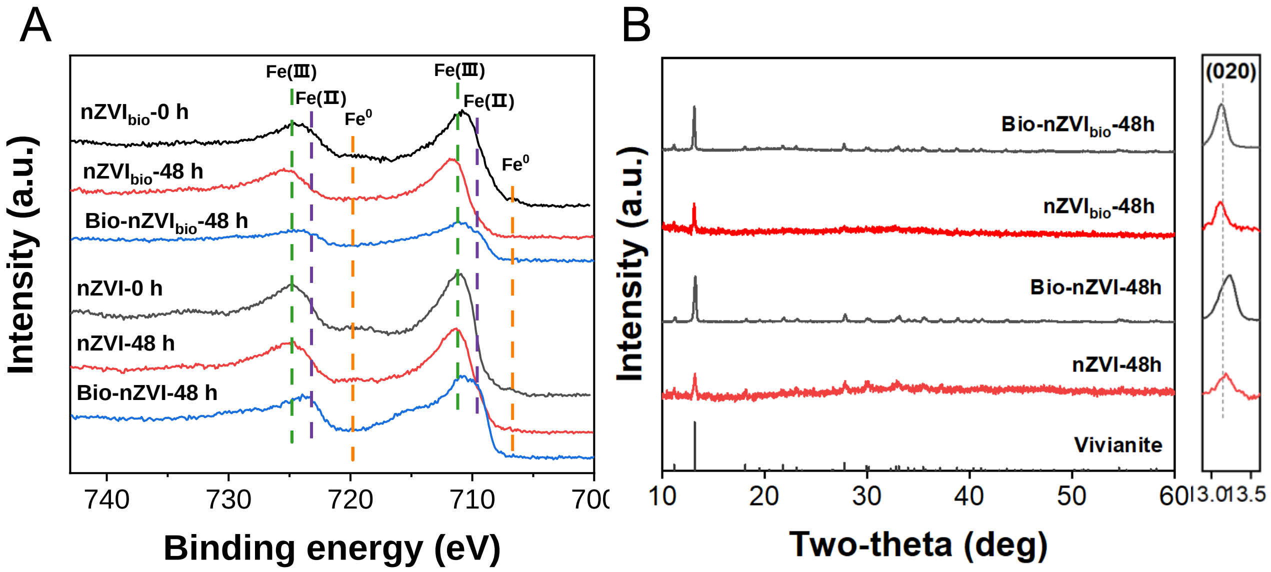

Fig. S11. XPS and XRD characterization of nanomaterials before and after reaction. (**A**) XPS spectra of Fe 2p for $nZVI_{bio}$ and nZVI before and after a 48-hour reaction. (**B**) XRD patterns of $nZVI_{bio}$ and nZVI after a 48-hour reaction.

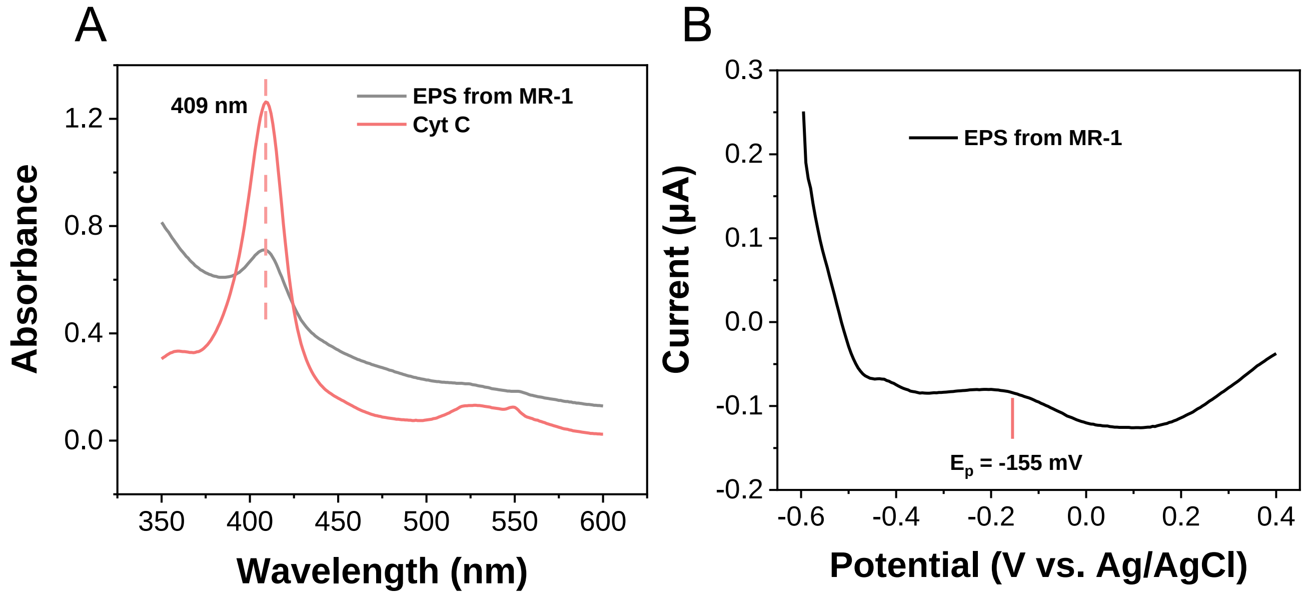

Fig. S12. Characterization of EPS from *S. oneidensis* MR-1. (**A**) UV-Vis spectra of *S. oneidensis* MR-1 EPS and cyt C. (**B**) DPV of EPS extracted from MR-1.

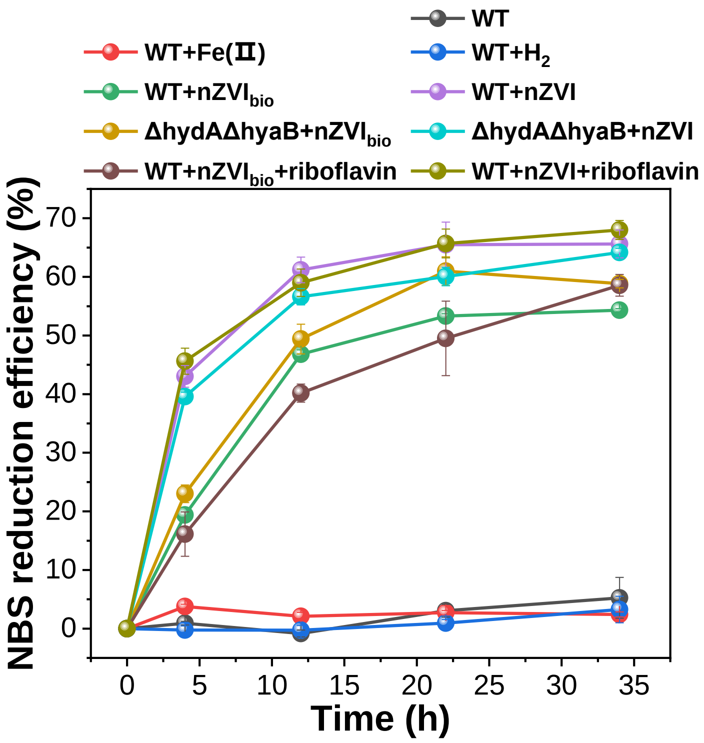

Fig. S13. Changes in the reduction efficiency of NBS over time with various strains coupled with Fe(II), H_2, riboflavin, $nZVI_{bio}$ or nZVI. WT: wild-type alone; WT + Fe(II): wild-type coupled with Fe(II); WT + H_2: wild-type coupled with H_2; WT + $nZVI_{bio}$: wild-type coupled with $nZVI_{bio}$; ΔhydAΔhyaB + $nZVI_{bio}$: hydrogenase knockout strain coupled with $nZVI_{bio}$;_WT + $nZVI_{bio}$ + riboflavin: wild-type coupled with $nZVI_{bio}$ and riboflavin.

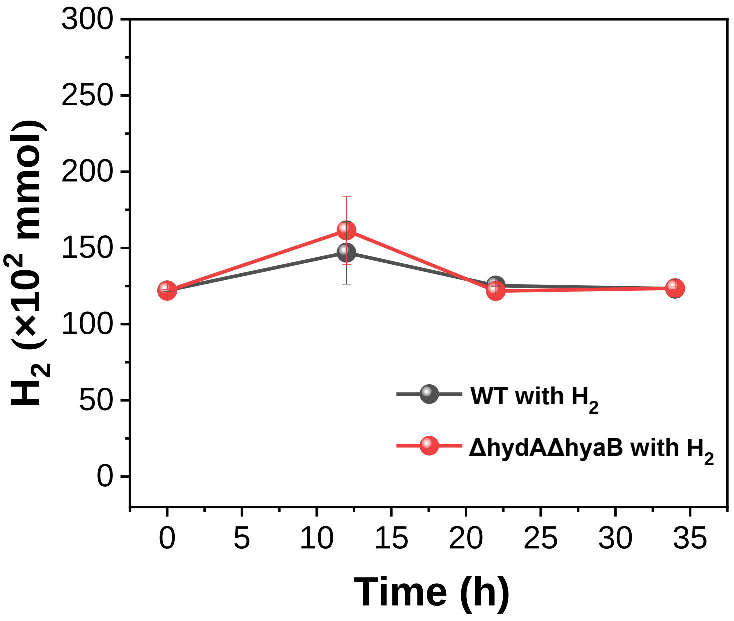

Fig. S14. Monitoring changes in H_2 content in the headspace of serum bottles containing either the wild-type or ΔhydAΔhyaB strain.

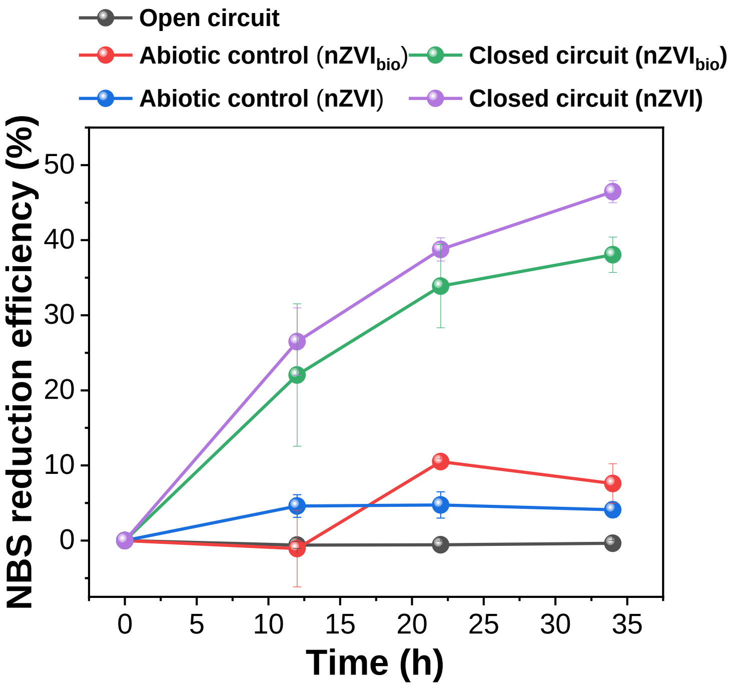

Fig. S15. Variations in the reduction efficiency of NBS over time within the abiotic group, the open circuit group, and the closed circuit group in the two-chamber galvanic cell.

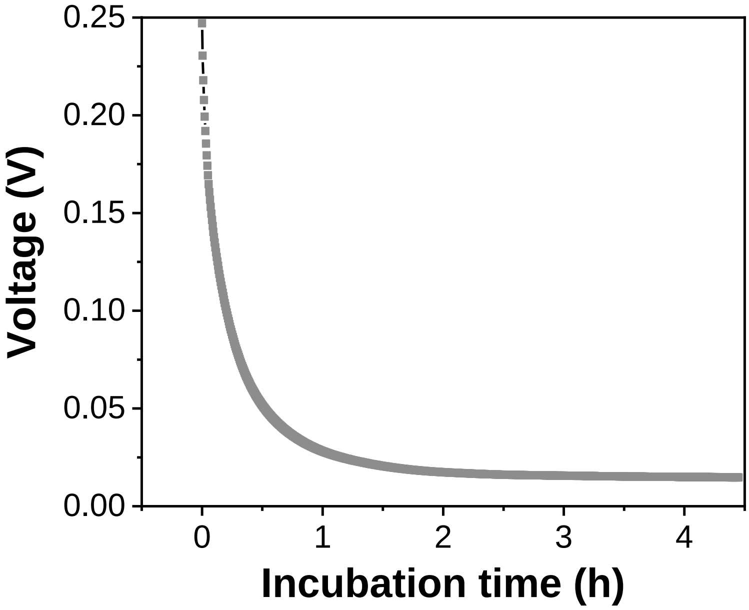

Fig. S16. The voltage monitoring of the galvanic cell after bacterial incubation.

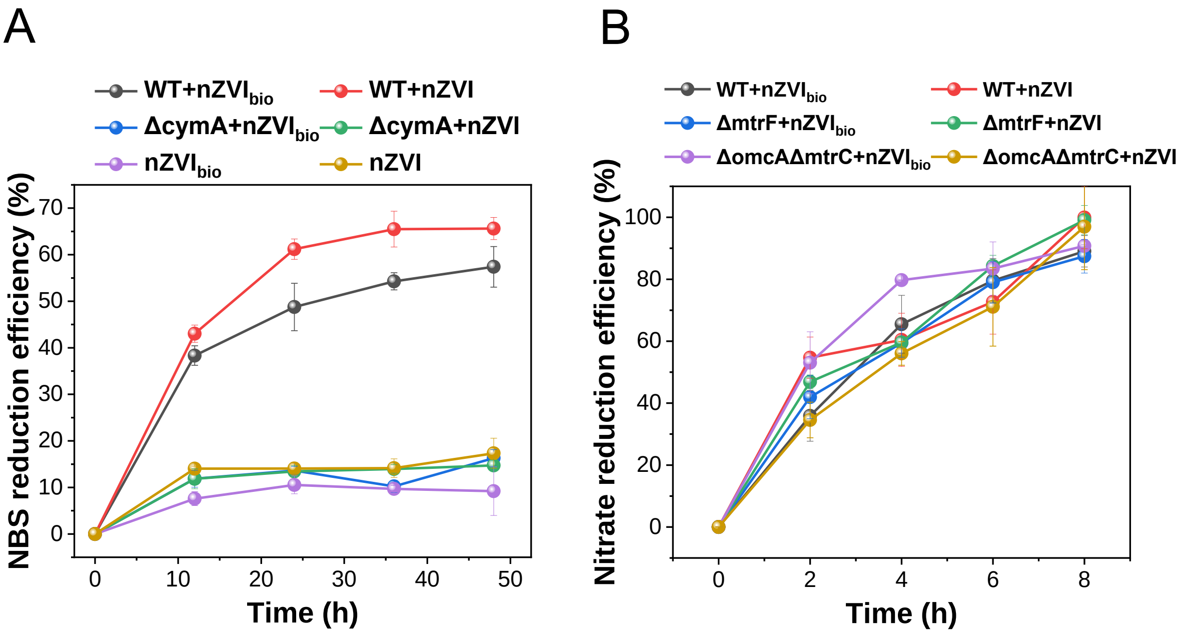

Fig. S17. NBS or nitrate reduction efficiency of different strains coupled with $nZVI_{bio}$ or nZVI. (**A**) Alteration in the reduction efficiency of NBS by nanomaterials, wild-type strain coupled with nanomaterials, or ΔcymA strain coupled with nanomaterials over time. (**B**) Variation in the reduction efficiency of nitrate by different strains coupled with $nZVI_{bio}$ or nZVI over time.

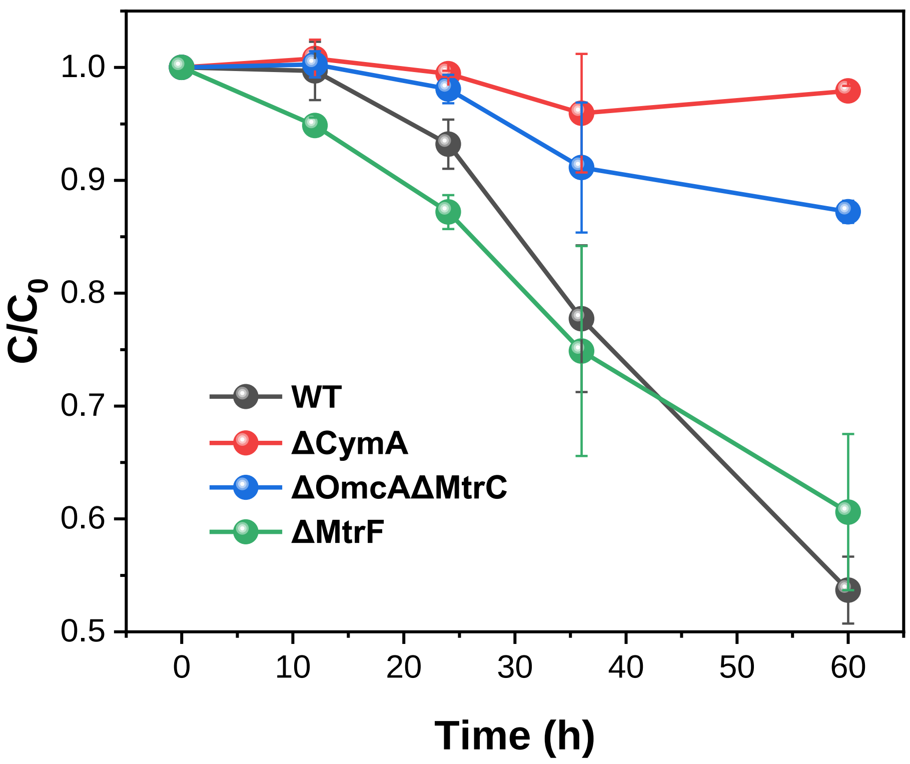

Fig. S18. NBS reduction by the wild-type, ΔcymA, ΔmtrF, or ΔomcAΔmtrC strain.

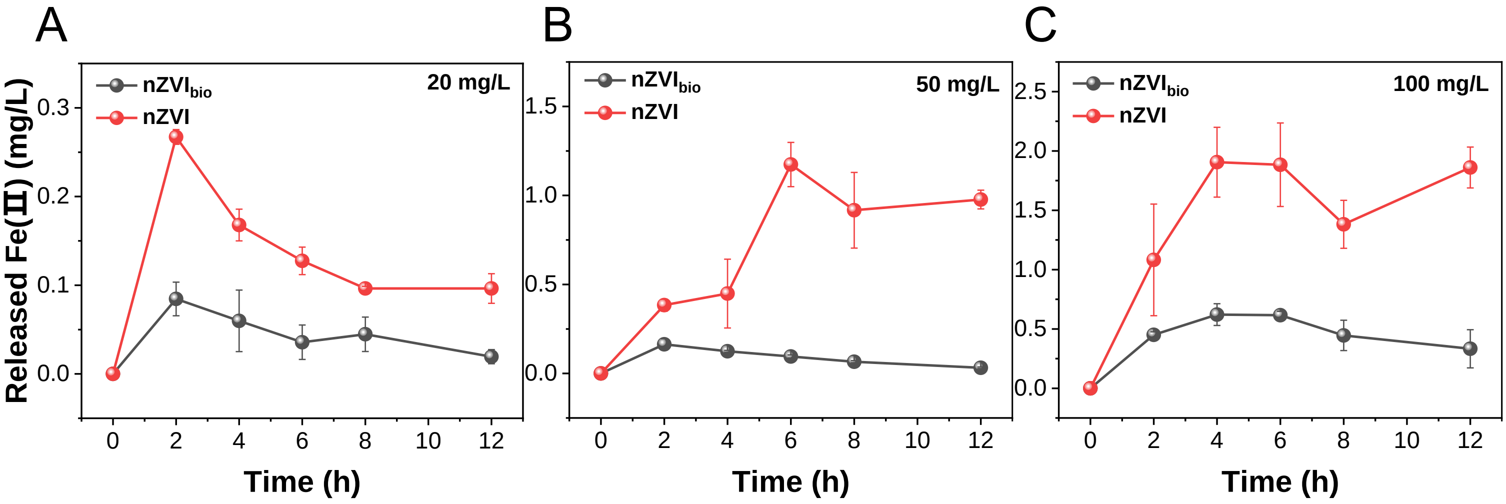

Fig. 19. Concentrations of released Fe(II) in the bio-nano systems with nZVI or $nZVI_{bio}$ at concentrations of 20 mg/L, 50 mg/L and 100 mg/L.

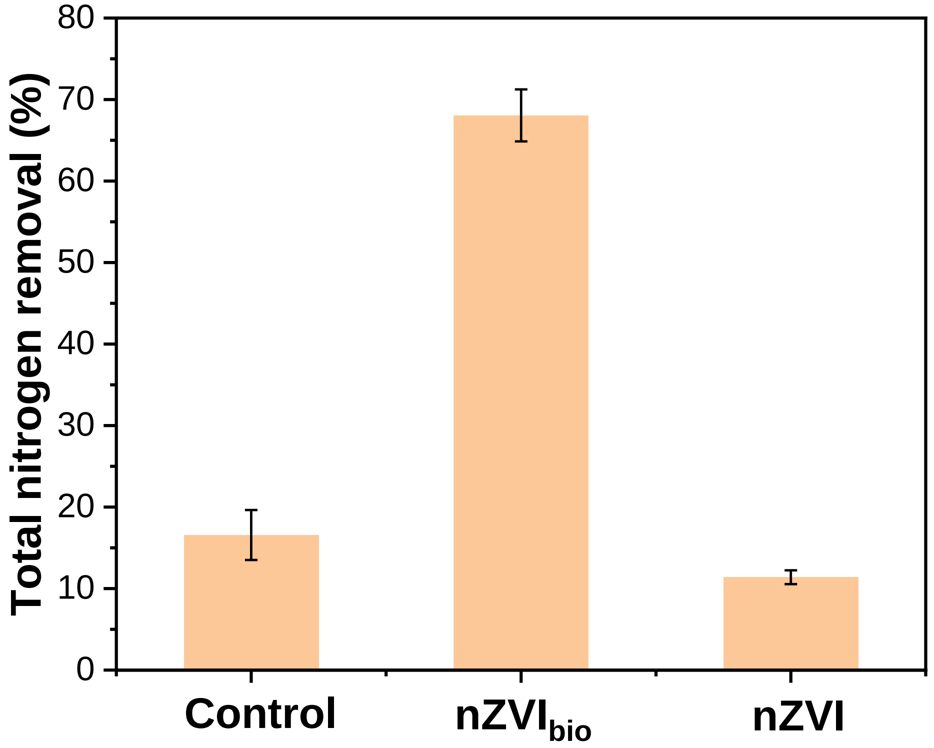

Fig. S20. Total nitrogen removal efficiencies of the denitrifying bacteria, bio-nZVI system and bio-nZVI$_{bio}$ system after treatment for 24 h.

Table S1. Differentially expressed genes of clustering heatmap.

Gene name	Function	Log_2FC ($nZVI_{bio}$ vs Control)	Log_2FC (nZVI vs Control)
ahpC	peroxiredoxin && PF10417:C-terminal domain of 1-Cys peroxiredoxin|PF00578:AhpC/TSA family	-0.29862	2.5299
ahpF	alkyl hydroperoxide reductase subunit F && PF13192:Thioredoxin domain|PF07992:Pyridine nucleotide-disulphide oxidoreductase	-0.12551	1.78531
katB	catalase KatB && PF00199:Catalase|PF06628:Catalase-related immune-responsive	-0.01866	2.47928
oxyR	hydrogen peroxide-inducible genes transcriptional activator OxyR && PF00126:Bacterial regulatory helix-turn-helix protein, lysR family|PF03466:LysR substrate binding domain	-0.02355	0.48659
SO_RS20800	cytochrome b/b6 domain-containing protein && PF01292:Prokaryotic cytochrome b561	3.190084	4.769991
SO_RS20765	Spy/CpxP family protein refolding chaperone && PF07813:LTXXQ motif family protein	4.014558	5.041951
pspA	phage shock protein PspA && PF04012:PspA/IM30 family	1.29071	1.63556
pspB	envelope stress response membrane protein PspB && PF06667:Phage shock protein B	1.16666	1.51313
pspC	envelope stress response membrane protein PspC && PF04024:PspC domain	1.15619	1.43332
zapD	cell division protein ZapD && PF07072:Protein of unknown function (DUF1342)	0.20767	-0.4946
zapA	cell division protein ZapA && PF05164:Cell division protein ZapA	0.58914	-0.06561
dnaA	chromosomal replication initiator protein DnaA && PF08299:Bacterial dnaA protein helix-turn-helix|PF00308:Bacterial dnaA protein|PF11638:DnaA N-terminal domain	-0.10168	-0.76665
dprA	DNA-processing protein DprA && PF02481:DNA recombination-mediator protein A	-0.36671	-0.85401
parC	DNA topoisomerase IV subunit A && PF00521:DNA gyrase/topoisomerase IV, subunit A	-0.30959	-0.5064
rpmH	50S ribosomal protein L34 && PF00468:Ribosomal protein L34	-0.67002	-2.7168
rpsC	30S ribosomal protein S3 && -	-0.31516	-1.5628
rpsT	30S ribosomal protein S20 && PF01649:Ribosomal protein S20	-0.37439	-1.60539
rpsB	30S ribosomal protein S2 && PF00318:Ribosomal protein S2	-0.24698	-1.13234
rplC	50S ribosomal protein L3 && PF00297:Ribosomal protein L3	-0.14091	-1.18368
rplY	50S ribosomal protein L25 && PF01386:Ribosomal L25p family	0.516461	-0.90001
rpsU	30S ribosomal protein S21 && PF01165:Ribosomal protein S21	-0.16259	-1.1471
cysD	sulfate adenylyltransferase subunit CysD && PF01507:Phosphoadenosine phosphosulfate reductase family	0.742032	1.301406
cysW	sulfate ABC transporter permease subunit CysW && PF00528:Binding-protein-dependent transport system inner membrane component	0.628195	1.088756
cysI	assimilatory sulfite reductase (NADPH) hemoprotein subunit && PF01077:Nitrite and sulphite reductase 4Fe-4S domain|PF03460:Nitrite/Sulfite reductase ferredoxin-like half domain	0.555867	1.04531

cysP	thiosulfate ABC transporter substrate-binding protein CysP && PF13531:Bacterial extracellular solute-binding protein	0.63844	1.047438
cysN	sulfate adenylyltransferase subunit CysN && PF00009:Elongation factor Tu GTP binding domain	0.412128	0.803748
cysK	cysteine synthase A && PF00291:Pyridoxal-phosphate dependent enzyme	0.457768	0.838636
atpB	F0F1 ATP synthase subunit A && PF00119:ATP synthase A chain	-0.16976	-1.5842
atpC	F0F1 ATP synthase subunit epsilon && PF02823:ATP synthase, Delta/Epsilon chain, beta-sandwich domain|PF00401:ATP synthase, Delta/Epsilon chain, long alpha-helix domain	-0.68564	-1.75444
atpD	F0F1 ATP synthase subunit beta && PF00006:ATP synthase alpha/beta family, nucleotide-binding domain|PF02874:ATP synthase alpha/beta family, beta-barrel domain|PF00306:ATP synthase alpha/beta chain, C terminal domain	-0.3848	-0.74398
atpG	F0F1 ATP synthase subunit gamma && PF00231:ATP synthase	-0.59679	-1.27138
atpH	F0F1 ATP synthase subunit delta && PF00213:ATP synthase delta (OSCP) subunit	-0.42912	-1.00252

REFERENCES AND NOTES

1. B. E. Logan, K. Rabaey, Conversion of wastes into bioelectricity and chemicals by using microbial electrochemical technologies. *Science* **337**, 686–690 (2012).

2. K. Rabaey, R. A. Rozendal, Microbial electrosynthesis—Revisiting the electrical route for microbial production. *Nat. Rev. Microbiol.* **8**, 706–716 (2010).

3. J. Keasling, H. Garcia Martin, T. S. Lee, A. Mukhopadhyay, S. W. Singer, E. Sundstrom, Microbial production of advanced biofuels. *Nat. Rev. Microbiol.* **19**, 701–715 (2021).

4. N. Kornienko, J. Z. Zhang, K. K. Sakimoto, P. Yang, E. Reisner, Interfacing nature's catalytic machinery with synthetic materials for semi-artificial photosynthesis. *Nat. Nanotechnol.* **13**, 890–899 (2018).

5. R. H. Stauber, S. Siemer, S. Becker, G.-B. Ding, S. Strieth, S. K. Knauer, Small meets smaller: Effects of nanomaterials on microbial biology, pathology, and ecology. *ACS Nano* **12**, 6351–6359 (2018).

6. Q. Zhao, S. Wang, Z. Lv, A. Zupanic, S. Guo, Q. Zhao, L. Jiang, Y. Yu, Using nanomaterials to increase the efficiency of chemical production in microbial cell factories: A comprehensive review. *Biotechnol. Adv.* **59**, 107982 (2022).

7. X. Gong, D. Huang, Y. Liu, Z. Peng, G. Zeng, P. Xu, M. Cheng, R. Wang, J. Wan, Remediation of contaminated soils by biotechnology with nanomaterials: Bio-behavior, applications, and perspectives. *Crit. Rev. Biotechnol.* **38**, 455–468 (2017).

8. R. Mukherjee, R. Kumar, A. Sinha, Y. Lama, A. K. Saha, A review on synthesis, characterization, and applications of nano zero valent iron (nZVI) for environmental remediation. *Crit. Rev. Environ. Sci. Technol.* **46**, 443–466 (2015).

9. S. Cestellos-Blanco, H. Zhang, J. M. Kim, Y.-X. Shen, P. Yang, Photosynthetic semiconductor biohybrids for solar-driven biocatalysis. *Nat. Catal.* **3**, 245–255 (2020).

10. Q. Xin, H. Shah, A. Nawaz, W. Xie, M. Z. Akram, A. Batool, L. Tian, S. U. Jan, R. Boddula, B. Guo, Q. Liu, J. R. Gong, Antibacterial carbon-based nanomaterials. *Adv. Mater.* **31**, 1804838 (2018).

11. H. Sun, J. Wang, Y. Jiang, W. Shen, F. Jia, S. Wang, X. Liao, L. Zhang, Rapid aerobic inactivation and facile removal of *Escherichia coli* with amorphous zero-valent iron microspheres: Indispensable roles of reactive oxygen species and iron corrosion products. *Environ. Sci. Technol.* **53**, 3707–3717 (2019).

12. S.-F. Hsieh, D. Bello, D. F. Schmidt, A. K. Pal, A. Stella, J. A. Isaacs, E. J. Rogers, Mapping the biological oxidative damage of engineered nanomaterials. *Small* **9**, 1853–1865 (2013).

13. F. Kang, P. J. Alvarez, D. Zhu, Microbial extracellular polymeric substances reduce Ag^{+} to silver nanoparticles and antagonize bactericidal activity. *Environ. Sci. Technol.* **48**, 316–322 (2014).

14. X. Han, Z. Wang, M. Chen, X. Zhang, C. Y. Tang, Z. Wu, Acute responses of microorganisms from membrane bioreactors in the presence of NaOCl: Protective mechanisms of extracellular polymeric substances. *Environ. Sci. Technol.* **51**, 3233–3241 (2017).

15. I. D. S. Henriques, N. G. Love, The role of extracellular polymeric substances in the toxicity response of activated sludge bacteria to chemical toxins. *Water Res.* **41**, 4177–4185 (2007).

16. G. P. Sheng, H. Q. Yu, X. Y. Li, Extracellular polymeric substances (EPS) of microbial aggregates in biological wastewater treatment systems: A review. *Biotechnol. Adv.* **28**, 882–894 (2010).

17. N. Zhu, S. Wang, C. Tang, P. Duan, L. Yao, J. Tang, P. K. Wong, T. An, D. D. Dionysiou, Y. Wu, Protection mechanisms of periphytic biofilm to photocatalytic nanoparticle exposure. *Environ. Sci. Technol.* **53**, 1585–1594 (2019).

18. L. Natarajan, M. A. Jenifer, A. Mukherjee, Eco-corona formation on the nanomaterials in the aquatic systems lessens their toxic impact: A comprehensive review. *Environ. Res.* **194**, 110669 (2021).

19. A. S. Adeleye, J. R. Conway, T. Perez, P. Rutten, A. A. Keller, Influence of extracellular polymeric substances on the long-term fate, dissolution, and speciation of copper-based nanoparticles. *Environ. Sci. Technol.* **48**, 12561–12568 (2014).

20. T. Liu, X. Luo, Y. Wu, J. R. Reinfelder, X. Yuan, X. Li, D. Chen, F. Li, Extracellular electron shuttling mediated by soluble c-type cytochromes produced by *Shewanella oneidensis* MR-1. *Environ. Sci. Technol.* **54**, 10577–10587 (2020).

21. Y. Xiao, E. Zhang, J. Zhang, Y. Dai, Z. Yang, H. E. M. Christensen, J. Ulstrup, F. Zhao, Extracellular polymeric substances are transient media for microbial extracellular electron transfer. *Sci. Adv.* **3**, e1700623 (2017).

22. F.-H. Li, Q. Tang, Y.-Y. Fan, H.-Q. Yu, Developing a population-state decision system for intelligently reprogramming extracellular electron transfer in *Shewanella oneidensis*. *Proc. Natl. Acad. Sci. U.S.A.* **117**, 23001–23010 (2020).

23. S. K. Springthorpe, C. M. Dundas, B. K. Keitz, Microbial reduction of metal-organic frameworks enables synergistic chromium removal. *Nat. Commun.* **10**, 5212 (2019).

24. J. Xu, A. Avellan, H. Li, X. Liu, V. Noël, Z. Lou, Y. Wang, R. Kaegi, G. Henkelman, G. V. Lowry, Sulfur loading and speciation control the hydrophobicity, electron transfer, reactivity, and selectivity of sulfidized nanoscale zerovalent iron. *Adv. Mater.* **32**, e1906910 (2020).

25. Y. Hu, X. Peng, Z. H. Ai, F. L. Jia, L. Z. Zhang, Liquid nitrogen activation of zero-valent iron and its enhanced Cr(VI) removal performance. *Environ. Sci. Technol.* **53**, 8333–8341 (2019).

26. J. Xu, Z. Cao, H. Zhou, Z. Lou, Y. Wang, X. Xu, G. V. Lowry, Sulfur dose and sulfidation time affect reactivity and selectivity of post-sulfidized nanoscale zerovalent iron. *Environ. Sci. Technol.* **53**, 13344–13352 (2019).

27. S. Bose, M. F. Hochella Jr., Y. A. Gorby, D. W. Kennedy, D. E. McCready, A. S. Madden, B. H. Lower, Bioreduction of hematite nanoparticles by the dissimilatory iron reducing bacterium *Shewanella oneidensis* MR-1. *Geochim. Cosmochim. Acta* **73**, 962–976 (2009).

28. P. Wilfert, A. I. Dugulan, K. Goubitz, L. Korving, G. J. Witkamp, M. C. M. V. Loosdrecht, Vivianite as the main phosphate mineral in digested sewage sludge and its role for phosphate recovery. *Water Res.* **144**, 312–321 (2018).

29. C. Yang, H. Aslan, P. Zhang, S. Zhu, Y. Xiao, L. Chen, N. Khan, T. Boesen, Y. Wang, Y. Liu, L. Wang, Y. Sun, Y. Feng, F. Besenbacher, F. Zhao, M. Yu, Carbon dots-fed *Shewanella oneidensis* MR-1 for bioelectricity enhancement. *Nat. Commun.* **11**, 1379 (2020).

30. J. Xiong, D. Chan, X. Guo, F. Chang, M. Chen, Q. Wang, X. Song, C. Wu, Hydrogen production driven by formate oxidation in *Shewanella oneidensis* MR-1. *Appl. Microbiol. Biotechnol.* **104**, 5579–5591 (2020).

31. J. M. V. Makabenta, A. Nabawy, C.-H. Li, S. Schmidt-Malan, R. Patel, V. M. Rotello, Nanomaterial-based therapeutics for antibiotic-resistant bacterial infections. *Nat. Rev. Microbiol.* **19**, 23–36 (2020).

32. Y. Hu, J. Wang, H. Sun, S. Wang, X. Liao, J. Wang, T. An, Roles of extracellular polymeric substances in the bactericidal effect of nanoscale zero-valent iron: Trade-offs between physical disruption and oxidative damage. *Environ. Sci. Nano* **6**, 2061–2073 (2019).

33. A. B. Djurišić, Y. H. Leung, A. M. C. Ng, X. Y. Xu, P. K. H. Lee, N. Degger, R. S. S. Wu, Toxicity of metal oxide nanoparticles mechanisms characterization and avoiding experimental artefacts. *Small* **11**, 26–44 (2015).

34. A. M. Mitchell, T. J. Silhavy, Envelope stress responses: Balancing damage repair and toxicity. *Nat. Rev. Microbiol.* **17**, 417–428 (2019).

35. A. J. Darwin, The phage-shock-protein response. *Mol. Microbiol.* **57**, 621–628 (2005).

36. S.-K. Sun, X. Xu, Z. Tang, Z. Tang, X.-Y. Huang, M. Wirtz, R. Hell, F.-J. Zhao, A molecular switch in sulfur metabolism to reduce arsenic and enrich selenium in rice grain. *Nat. Commun.* **12**, 1392 (2021).

37. C. Andrés-Barrao, H. Alzubaidy, R. Jalal, K. G. Mariappan, A. Zélicourt, A. Bokhari, O. Artyukh, K. Alwutayd, A. Rawat, K. Shekhawat, M. Almeida-Trapp, M. M. Saad, H. Hirt, Coordinated bacterial and plant sulfur metabolism in *Enterobacter* sp. SA187–induced plant salt stress tolerance. *Proc. Natl. Acad. Sci. U.S.A.* **118**, e2107417118 (2021).

38. E. Roach, "Investigation of the structures of ZapA and ZapD from *Escherichia coli* and their roles in bacterial cell division," thesis, University of Guelph, Guelph, Ontario (2015).

39. M. Ventroux, M. F. Noirot-Gros, Prophage-encoded small protein YqaH counteracts the activities of the replication initiator DnaA in *Bacillus subtilis*. *Microbiology* **168**, 001268 (2022).

40. S. W. Li, G. P. Sheng, Y. Y. Cheng, H. Q. Yu, Redox properties of extracellular polymeric substances (EPS) from electroactive bacteria. *Sci. Rep.* **6**, 39098 (2016).

41. L. Shi, H. Dong, G. Reguera, H. Beyenal, A. Lu, J. Liu, H.-Q. Yu, J. K. Fredrickson, Extracellular electron transfer mechanisms between microorganisms and minerals. *Nat. Rev. Microbiol.* **14**, 651–662 (2016).

42. H. W. Harris, M. Y. El-Naggar, O. Bretschger, M. J. Ward, M. F. Romine, A. Y. Obraztsova, K. H. Nealson, Electrokinesis is a microbial behavior that requires extracellular electron transport. *Proc. Natl. Acad. Sci. U.S.A.* **107**, 326–331 (2010).

43. A. Okamoto, K. Hashimoto, K. H. Nealson, R. Nakamura, Rate enhancement of bacterial extracellular electron transport involves bound flavin semiquinones. *Proc. Natl. Acad. Sci. U.S.A.* **110**, 7856–7861 (2013).

44. X. Li, J. Li, X.-R. Yu, Y.-K. Zhu, H.-Q. Liu, L. Chen, J. Wu, X.-Z. Fu, S. Cui, T. Y. Huang, R.-Q. Ye, W.-W. Li, Zero-valent iron boosts nitrate-to-ammonia bioconversion via extracellular electron donation and reduction pathway complementation. *Resour. Conserv. Recycl.* **188**, 106687 (2023).

45. Q. Wang, A.-A. D. Jones III, J. A. Gralnick, L. Lin, C. R. Buie, Microfluidic dielectrophoresis illuminates the relationship between microbial cell envelope polarizability and electrochemical activity. *Sci. Adv.* **5**, eaat5664 (2019).

46. Z. Li, K. Greden, P. J. Alvarez, K. Gregory, G. V. Lowry, Adsorbed polymer and NOM limits adhesion and toxicity of nano scale zerovalent iron to *E. coli*. *Environ. Sci. Technol.* **44**, 3462–3467 (2010).

47. H. M. Ibrahim, M. Awad, A. S. Al-Farraj, A. M. Al-Turki, Stability and dynamic aggregation of bare and stabilized zero-valent iron nanoparticles under variable solution chemistry. *Nanomaterials* **10**, 192 (2020).

48. T. Raychoudhury, N. Tufenkji, S. Ghoshal, Aggregation and deposition kinetics of carboxymethyl cellulose-modified zero-valent iron nanoparticles in porous media. *Water Res.* **46**, 1735–1744 (2012).

49. R. H. Fang, W. Gao, L. Zhang, Targeting drugs to tumours using cell membrane-coated nanoparticles. *Nat. Rev. Clin. Oncol.* **20**, 33–48 (2023).

50. M. Mahmoudi, M. P. Landry, A. Moore, R. Coreas, The protein corona from nanomedicine to environmental science. *Nat. Rev. Mater.* **8**, 422–438 (2023).

51. S. B. Pereira, A. Sousa, M. Santos, M. Araújo, F. Serôdio, P. Granja, P. Tamagnini, Strategies to obtain designer polymers based on cyanobacterial extracellular polymeric substances (EPS). *Int. J. Mol. Sci.* **20**, 5693 (2019).

52. B. B. Wang, X. T. Liu, J. M. Chen, D. C. Peng, F. He, Composition and functional group characterization of extracellular polymeric substances (EPS) in activated sludge: The impacts of polymerization degree of proteinaceous substrates. *Water Res.* **129**, 133–142 (2018).

53. G. Zhu, Z. Xu, L. T. Yan, Entropy at bio-nano interfaces. *Nano Lett.* **20**, 5616–5624 (2020).

54. W. Gao, R. H. Fang, S. Thamphiwatana, B. T. Luk, J. Li, P. Angsantikul, Q. Zhang, C.-M. J. Hu, L. Zhang, Modulating antibacterial immunity via bacterial membrane-coated nanoparticles. *Nano Lett.* **15**, 1403–1409 (2015).

55. K. K. Sakimoto., A. B. Wong., P. Yang., Self-photosensitization of nonphotosynthetic bacteria for solar-to-chemical production. *Science* **351**, 74–77 (2016).

56. Y. Deng, Z. Li, R. Tang, K. Ouyang, C. Liao, Y. Fang, C. Ding, L. Yang, L. Su, D. Gong, What will happen when microorganisms "meet" photocatalysts and photocatalysis? *Environ. Sci. Nano* **7**, 702–723 (2020).

57. S. Jin, Y. Jeon, M. S. Jeon, J. Shin, Y. Song, S. Kang, J. Bae, S. Cho, J.-K. Lee, D. R. Kim, B.-K. Cho, Acetogenic bacteria utilize light-driven electrons as an energy source for autotrophic growth. *Proc. Natl. Acad. Sci. U.S.A.* **118**, e2020552118 (2021).

58. D.-F. Liu, D. Min, L. Cheng, F. Zhang, D.-B. Li, X. Xiao, G.-P. Sheng, H.-Q. Yu, Anaerobic reduction of 2,6-dinitrotoluene by *Shewanella oneidensis* MR-1: Roles of Mtr respiratory pathway and NfnB. *Biotechnol. Bioeng.* **114**, 761–768 (2017).

59. P. J. L. Simpson, D. J. Richardson, R. Codd, The periplasmic nitrate reductase in *Shewanella*: The resolution, distribution and functional implications of two NAP isoforms, NapEDABC and NapDAGHB, *Microbiology* **156**, 302–312 (2010).

60. B. Cao, Z. Zhao, L. Peng, H.-Y. Shiu, M. Ding, F. Song, X. Guan, C. K. Lee, J. Huang, D. Zhu, X. Fu, G. C. L. Wong, C. Liu, K. Nealson, P. S. Weiss, X. Duan, Y. Huang, Silver nanoparticles boost charge-extraction efficiency in *Shewanella* microbial fuel cells. *Science* **373**, 1336–1340 (2021).
